# Improved identification of pollution source attribution by using PAH ratios combined with multivariate statistics

**DOI:** 10.1038/s41598-022-23966-4

**Published:** 2022-11-11

**Authors:** Matilda Mali, Rosa Ragone, Maria Michela Dell’Anna, Giuseppe Romanazzi, Leonardo Damiani, Piero Mastrorilli

**Affiliations:** DICATECh, Politecnico di Bari, Via Orabona, 4, 70125 Bari, Italy

**Keywords:** Biogeochemistry, Environmental sciences, Risk factors, Chemistry

## Abstract

Polycyclic aromatic hydrocarbons (PAHs) are contaminants introduced by different pathways in the marine ecosystem, affecting both aquatic and sediment bodies. Identification of their sources is of vital importance for protecting the marine ecosystem. The attribution of the pollution sources is usually made by using diagnostic molecular ratios of PAHs isomers. The reliability of this approach diminishes when PAHs contents are measured far from their original source, for example in water bodies or in bottom sediments. Conventionally the source attribution is based on time consuming univariate methods. In the present work coupling of molecular ratios with advanced supervised statistical techniques was used to increase the accuracy of the PAH source attribution in bottom sediments. Data on PAHs distribution within 5 port areas, with known pattern port activity, were collected. Evaluation of multiple PAHs ratios at once by means of supervised OPLS-DA technique was performed. A robust descriptive and predictive model was set up and successfully validated. The proposed methodology helps identify PAH transport pathways, highlighting interactions between pollution patterns, port activities and coastal land-use supporting decision makers in defining monitoring and mitigation procedures.

## Introduction

Polynuclear aromatic hydrocarbons (PAHs) are among the major contaminant categories investigated in the marine environment due to their high toxicity and environmental persistence. The carcinogenic and mutagenic properties of PAHs have driven their designation as persistent organic pollutants (POPs) by the UNECE Convention on Long-range Transboundary Air Pollution^1^ (CLRTAP 1979) and their listing as priority substances by OSPAR Convention and Water Framework Directive 2000/60/EC^2,3^. Among several hundreds of individual PAHs, 16 congeners have been selected as priority pollutants by the United States Environmental Protection Agency (USEPA), based on their toxicity, ease of analysis, and environmental occurrence with time^4^.

PAHs are abundant in the atmosphere and can be easily introduced in the marine ecosystem in both aquatic and sediment media^5,6^. Different anthropogenic activities occurring on land or coastal and port areas, such as dry-docking operations, loading and unloading of bulk freights, discharging of bilge oil or wastewater urban discharges, and washout of airborne particles (soot), are responsible for PAHs contamination and their vehiculation from the terrestrial environment into marine ecosystems^7,8^.

PAHs are thermally very stable due to their molecular structure (i.e. delocalized π-electrons); thus, once introduced in marine areas they persist for a long time, entrapped in the bottom sediment that acts as a sink for such compounds^9^. In addition, in marine sediments, PAHs can also naturally occur, deriving from terrestrial plant waxes, marine phytoplankton and bacteria, and diagenetic transformation of biogenic precursors. Determination of PAHs concentration and identification of their dominant sources constitute crucial steps for optimizing the accuracy of risk assessment and consequent choice of mitigation measures. Distinction of different PAHs sources is generally based on the use of diagnostic molecular ratios, namely the relative proportions of specific PAH isomers^5,8,10–12^ The validity of this approach lies on the assumption that such ratios are specific for a given emission source and are preserved along transport pathway to the receptor, thus constituting a fingerprinting of the pollution type^6,13,14^. However, results obtained by means of diagnostic ratios can be significantly biased due to modifications occurring to PAHs when deposited on sediments. Indeed, PAHs pattern is influenced by different factors, such as climatic conditions, weathering and aging processes, anoxic conditions in the deeper sediment layers, photodegradation or microbial activity, presence of organic matter, as well as granulometry of sediments. The difficulties in distinguishing among different PAHs sources is well documented^5,6,14–22^. Conventionally the attribution of pollution sources is based on analysis of the diagnostic ratio’s values one at a time or at least in pairs. The mere use of single index for PAHs source attribution cannot be considered plentifully convincing; consequently, in-depth studies addressing the relative suitability of various commonly applied PAH ratios, as indicators of sediment contaminant spectrum, are still desirable.

The aim of the present study was to provide a comprehensive approach able to overcome the critical issues discussed above, by evaluating at once multiple PAHs ratios through multivariate techniques^23–25^ in order to set up robust statistical (descriptive and predictive) models, that improve the diagnostic accuracy of PAH ratios. In this way, a comprehensive picture of the PAH pollution phenomenon can be obtained in time-saving way.

Specifically, Principal components analysis (PCA) and Orthogonal Partial Least Squares-Discriminant Analysis (OPLS-DA) were combined with the following diagnostic molecular ratios: IP/(IP + B[ghi]P) (Indeno(1,2,3-c,d)Pyrene) to Benzo(g,h,i)Perylene); BaA/(BaA + Chr) (Benzo(a)Anthracene to Chrysene); BaP/(BaP + Chr) (Benzo(a)Pyrene to Chrysene); BaA/BaA + BeP (Benzo(a)Anthracene to Benzo(e)Pyrene); BbF/BbF + BkF (Benzo(b)Fluoranthene to Benzo(k)Fluoranthene); Phen/Phen + Anth (Phenanthrene to Anthracene) and other ratios between PAH congeners commonly used in literature^6,9–11,13^. Different marine and port areas of the Apulia Region (Southern Italy), subjected to anthropogenic pressures generating different PAHs pattern, were selected for the present study. Details on land-use and human induced activities for each area are reported in Appendix A in Supplementary Information (SI).

The workflow developed through the following steps: (i) recognition of the most discriminant PAHs pattern characterizing each area investigated; (ii) identification of pressures and activities influencing each considered area; (iii) comparison of different PAHs ratios associated to each pollution source; (iv) set up of robust classification models that improved the accuracy of the PAHs ratio in contamination source attribution; (v) validation of the statistical model by using internal and external datasets as prediction sets.

## Materials and methods

### Geographical and environmental setting

**Figure 1:**
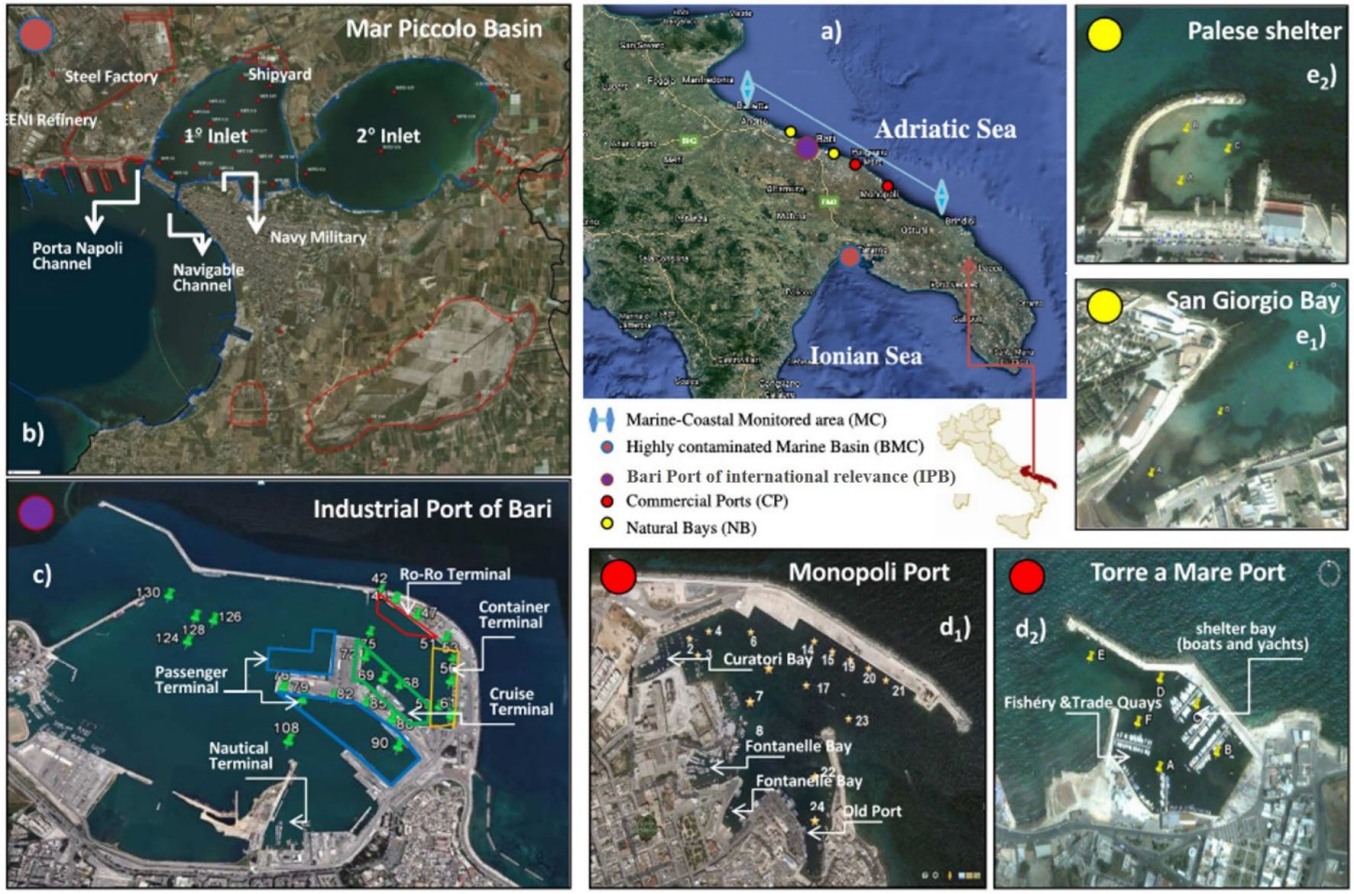
(**a**) Apulia coast in southeastern Italy with the indication of Marine Coastal (MC) Transect investigated (light blue arrow); (**b**) Mar Piccolo Basin and its main industrial pressures (BMC); (**c**) Port of Bari dealing with different port activities of an international relevance (IPB), (**d**_**1**_) Port of Monopoli (MP) and indication of the main internal bays; (**d**_**2**_) Port of Torre a Mare (TM) and the areas for port operations; (**e**_**1**_) Palese shelter (PL) and (**e**_**2**_) San Giorgio (SG) bay. The map was obtained through Google Earth Software (https://www.google.it/intl/it/earth/, Data SIO, NOAA, U.S.Navy, NGA, GEBCO © 2015 Google) and further modified with Power Point Software—License to Polytechnic University of Bari (Italy).

The study area stretches for 350 km along the shoreline between Adriatic and Ionian Seas, in Apulia (southeastern part of Italy) (Fig. 1). The dynamics of the Apulian coast depend on complex interactions between emerged and submerged morphological elements, hydrologic factors, climatic and sea-weather conditions, and human impacts^26^. Particularly in the last decades, human‐induced pressures strongly altered the dynamics of the coastal environment. The numerous port and harbors operating alongside the shoreline have progressively modified the coast morphology, consequently influencing the quality of the port basins. The ports are subjected to different activities (industrial, commercial, and touristic) that, along with activities occurring in the surrounding areas, have seriously affected the quality of port sediments^11,12,27–31^. It is worth noting that the sediment contaminant spectrum of a port reveals the impact of the port activity^32–38^. On these grounds, for the purpose of the present study, different harbors and coastal areas alongside both Adriatic and Ionian Sea were selected. The features of each specific area are schematically shown in Fig. 1 and specific details are reported in Appendix A in SI.

### Sampling

The sediment samples used in the present study were collected from May 2010 up to September 2011. The sampling was performed according to the national guidelines^39^ using continuous coring down to different depths. For each representative area, the sampled sites were selected according to the known anthropogenic pressure as described below, for a total of 78 sites and 158 sediment samples (see Appendix A for details). For the Mar Piccolo basin (BMC), 20 sites were selected mainly in the southern part of the first bay^35,40^ (Appendix A, Fig. S1), in the vicinity of the main pressure sources loading on the area (Channels, Navy, Steel Factory, ENI refinery). A core-sediment up to 3.0 m depth was employed; each core was divided into 50 cm length sub-cores for different 50 cm intervals that are marked with the letters (a), (b), (c), (d), (e) and (f) for the ranges 0–50 cm, 50–100 cm, 100–150 cm, 150–200 cm, 200–250 cm and 200–300 cm respectively. For the Port of Bari, classified as port with an international relevance (IPB), 23 sites were selected, sampling up to 2.0 m in depth (Fig. S2, the same lettering was adopted for the different sub-cores: “a” for 0–50 cm, “b” for 50–100 cm, “c” for 100–150 cm, and “d” for > 150 cm in depth); the sampled sites are representative of the main activities occurring within the port (terminals of passengers, containers, ro-ro and cruises). For the Commercial Ports (CP), 15 sites were sampled from the Port of Monopoli (MP) and 5 sites from the port of Torre a Mare (TM) (Fig. S3), all representative of the human-induced pressure loading on the basins. For the Natural Bays (NB), 3 sites in the Palese shelter (PL) and 3 sites in San Giorgio Bay (SG) were selected, with a coring depth reaching a maximum of 1.5 m (Fig. S4). For the Marine-Coastal area (MC), 9 sites were sampled in two marine transects (at 200 and 500 m from the coastline, Fig. S5), at shorter sediment core depths (45–60 cm long).

Sampling of sediments from the bottom-sea was carried out using vibro-corer PF1, equipped with a liner and a support vessel provided with differential GPS system for positioning of sampling cores. From each sub-cores of 50 cm length, aliquots of 0.500 kg of wet-sediment specimens were transferred from the liner into cleaned plastic bags; bags were held at 4 °C during transportation to laboratory and then stored at − 20 °C until the analyses.

### Analytical methods

Sixteen EPA PAHs were considered: naphthalene (Naph), anthracene (Ant), phenanthrene (Phen), acenaphthene (Ace), acenaphthylene (Acy), fluorene (Flu), fluoranthene (Flt), pyrene (Py), chrysene (Chr), benz(a)anthracene (BaA), benzo(a)pyrene (BaP), benzo(b)fluoranthene (B(b)F), benzo(k)fluoranthene (B(k)F), dibenzo(a,h)anthracene (DB(ah)A), benzo(g,h,i)perylene (B(ghi)P), and indeno(1,2,3-c,d)pyrene (IP). After specific clean-up^41,42^ PAHs were determined by gas chromatography coupled to mass spectrometry (GC–MS)^43^. The recovery rates, validated with spiked samples, ranged from 50% to Naph to 90% for Chr. PAHs were classified based on their molecular weight as Light PAHs (LPAHs) (< 200 and 2–3 rings) and Heavy PAHs (HPAHs) (> 200; more than 3 rings), and grouped on the basis of the number of the aromatic rings (2-, 3-, 4-, 5-, and 6-rings).

All analyses were carried out by certified agencies (ARPA-Apulian Regional Agency for Environmental Protection and ISPRA-Superior Institute for Protection and Environmental Research) according to highly standardized protocols^39^.

### PAH isomeric ratios

In the present study, the PAH isomeric ratios are expressed as the ratio of the thermodynamically most stable isomer (S) to the most unstable isomer (U) by using the formula: isomeric ratio = $\frac{S+U}{U}$. We adopted this formula because it gives smaller RSD, thus it is more suitable for multivariate analyses^44^ although the S/U form can be exploited for PAHs ratios due to the consistency of the relative standard deviations (RDS) of the S/U ratio that is usually constant and independent from the numerical values of S and U. However, values deriving from both S/(S + U) form and S/U form were considered during the discussion of results.

A total of 15 PAHs isomeric ratios were considered (Table S2). The typical six isomeric ratios BaA/(BaA + Chr), IP/(IP + B(ghi)P), BaP/(BaP + Chr), BaA/(BaA + BaP); Phen/(Phen + Anth), BbF/(BbF + BkF) were used to distinguish between pyrolytic from petrogenic sources. In addition, the following PAHs ratios were also calculated: the ratios of the sums of 2-, 3-, 4-, 5-, and 6-ringed congeners to the total Σ16PAHs and the ratios between some groups of congeners (LPAHs/tot, HPAH/tot, 2 + 3-rings/tot, where “tot” stands for Σ16PAHs) and 4-rings/5-rings, that can help identify specific sources. Finally, the sums of LPAHs and HPAHs separately were considered too^6,8,10,11,14,19,20,45–52^.

### Statistical analyses

Multivariate statistical analyses were performed by using SIMCA 16 Program (16, MKS Umetrics AB, Sweden). The 158 sediment samples, that constituted the observations, were grouped into five classes (BMC = Mar Piccolo sites, IPB = port of Bari classified as international relevance port, CP = commercial ports, NB = natural bays, and MC = marine-coastal samples), according to the main activities loading on each selected area (see “Sampling” section and Appendix A): BMC includes the Mar Piccolo Basin samples representative of industrial PAHs fingerprint; IPB includes the samples collected within the Port of Bari (BA), representative of a port dedicated to international maritime connections, thus of relevant contamination pattern; CP includes samples collected within the port of Monopoli (MP) and within the Port of Torre a Mare (TM), representative of commercial/touristic port pattern; NB includes the samples collected within two minor natural bays, Port of Palese (PL) and San Giorgio (SG) Bay thus representative of neglected port activity; finally, MC includes the samples collected on coastal marine transects representative of pristine PAH contamination.

A total of 33 variables was considered, consisting of the absolute values of concentration of the 16 above-mentioned PAHs and the values of 17 different diagnostic PAHs parameters (Tables S1, S2). As data pre-treatment, where the concentration resulted below the detection limit, half of the detection limit was used; then, all variables were scaled to unit-variance (UV) before statistical analysis. Unsupervised (Principal Component Analysis, PCA) and supervised (Orthogonal Partial Least Squares-Discriminant Analysis, OPLS-DA) approaches were applied. The quality of statistical models was evaluated based on *R*^2^ (goodness-of-fit), that expresses the fraction of the Sum of Squares (SS) explained by the model, and *Q*^2^ (goodness-of-prediction in cross-validation) that represents the fraction of the total variation of X or Y that can be predicted by a component, as estimated by cross-validation., computed according to the following Equation:

1 $${\text{Q}}^{{2}} = \, \left( {{1}.0 \, - {\text{ PRESS}}/{\text{SS}}} \right),$$

where PRESS is the Prediction Error Sum of Squares, i.e. the squared differences between observed and predicted values for the data kept out of the model fitting. Details on the cross-validation procedure are reported in the Appendix B in SI.

## Results and discussion

### PAHs distribution

The five study areas are subjected to different PAHs contamination levels. Specific information on PAHs distribution can be obtained by inspection of Table S1. The total concentration of PAHs (referred to as Σ16PAHs) ranged from 22.31 μg/kg (in MC) to 39,702 μg/kg (in BMC). Very high level of PAH contamination was logged in surficial sediments of BMC. On the contrary, the corresponding deepest strata, countersigned with “e” and “f” (at 3.80 m depth) were characterized by very low PAHs concentration (9.58 μg/kg).

**Figure 2:**
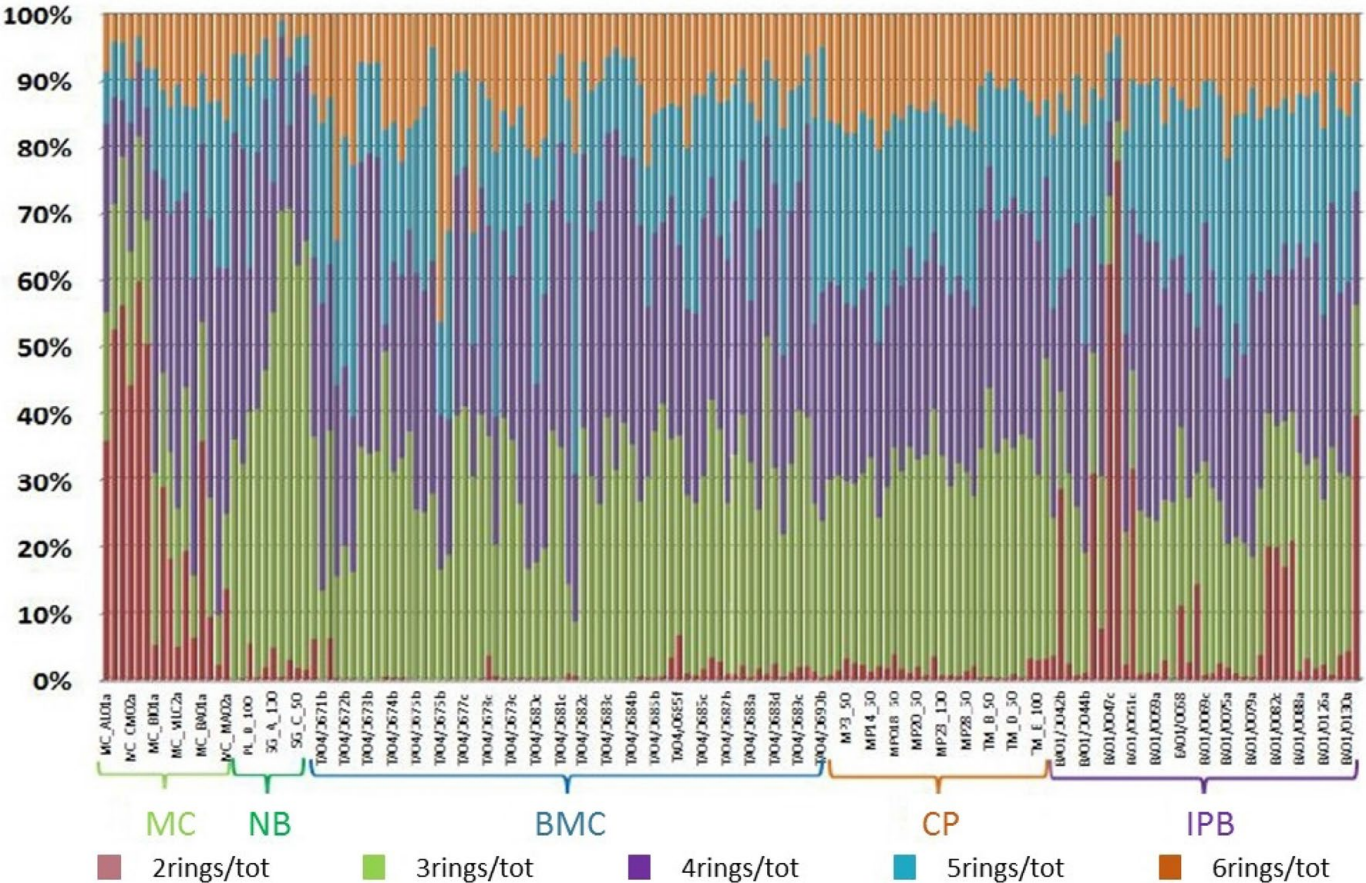
Percentage of 2-, 3-, 4-, 5-, and 6-ringed PAHs with respect to Σ16PAHs (where Σ16PAHs is indicated shortly as “tot”).

First information on PAHs sources can be obtained by the concentration level of compounds with different number of aromatic rings (Fig. 2). It is well-known that light PAHs (LPAHs, i.e. 2- and 3-rings) are abundant in petrogenic sources, while high molecular PAHs (HPAHs, i.e. 4-, 5-, and 6-rings) are indicative of pyrogenic origin^10^. In the investigated areas, the 3-ringed PAHs contributed to about 46% of Σ16PAHs in NB, ~ 30% in CP, ~ 27% in BMC, ~ 23% in IPB and ~ 16% in MC. The 4-ringed PAHs contributed to about 41% of Σ16PAHs in MC, ~ 38% in NB, ~ 30% in BMC, ~ 31% in IPB, and ~ 29% in CP. The 5- and 6-ringed congeners (BaP, BbF, BkF and DB(ah)A) varied from 43% of the Σ16PAHs in the BMC to 16% of the Σ16PAHs in the NB. In general, LPAHs contributed to 27% of Σ16PAHs in BMC, 28% of the Σ16PAHs in MC, 31–32% of Σ16PAHs in IPB and CP and 46% of Σ16PAHs in NB. HPAHs contributed to 73% of Σ16PAHs in BMC, 72% of the Σ16PAHs in MC, 68–69% of Σ16PAHs in IPB and CP and 54% of Σ16PAHs in NB. The 3- and 4-ringed PAHs are the most abundant PAHs in the NB, reaching 84% of Σ16PAHs. The 2-ringed PAH (i.e. naphthalene) reached the maximum values in MC (12% of Σ16PAHs) and IPB (7% of Σ16PAHs).

The pollution level of PAHs in sediments can be categorized into four types: absent or low (0–100 μg/kg Σ16PAHs), moderate (100–1000 μg/kg Σ16PAHs), high (1000–5000 μg/kg Σ16PAHs), and very high (> 5000 μg/kg Σ16PAHs)^53^. Low to moderate levels of PAHs were logged in the marine coastal (MC) sediments and in samples collected in PL and SG. On the contrary, BMC (Mar Piccolo), IPB (Bari) and CP (Monopoli and Torre a Mare) showed the highest values of Σ16PAHs (up to 39,702 μg/kg for BMC, up to 23,148 μg/kg in CP, and up to 18,046 μg/kg in IPB). Differences were registered also within the internal basins of a port: in fact, the samples located in the entrance of the port and in the deepest strata (> 2.00 m countersigned with "d") in Port of Bari (IPB) and those located in the deepest strata (> 3.00 m countersigned with "e" and "f") of the Mar Piccolo basin (BMC) showed the lowest Σ16PAHs concentrations. The obtained values give insights into the petrogenic/pyrogenic origin of the PAHs, nevertheless a deeper analysis is still required to correctly identify the PAHs sources regarding specific anthropogenic pressures.

### Multivariate statistical analyses

Generally, distinction of PAHs sources is based on the use of their diagnostic molecular ratios, considered singly or, at most, in pairs. We decided to use the absolute values of concentration of all the 16 above-mentioned PAHs (see “PAHs distribution” section) combined with 17 diagnostic PAHs parameters (15 isomeric ratios and 2 PAH sums, LPAHs and HPAHs, Table S2), as a dataset for the multivariate analysis. The multivariate analyses reduce the number of the original variables without losing important information, by constructing new variables called Principal Components (or Latent Components) that account for the largest variance of the original system, projected in a new hyper-space (i.e. statistical model). Each PC explains a portion of the variance existing among data; for a given PC, each observation has a score, indicating its contribution to the PC, while each original variable has a loading, indicating its weight on that PC.

First, the unsupervised Principal Component Analysis (PCA) was carried out to get a preliminary overview of data. Then, the supervised Orthogonal Partial Least Squares-Discriminant Analysis (OPLS-DA) was applied for an easier identification of those variables that mostly discriminated between the a priori set classes (here, the five studied areas).

#### PCA and OPLS-DA

A PCA model was built by using all observations (158 samples, collected in the five areas: BMC, CP, IPB, MC, and NB). The two first Principal Components, PC1 and PC2, explained together 52% of total variance (*R*^2^X[1] = 0.39; *R*^2^X[2] = 0.13) (Fig. S6).

Aiming at maximizing the differences between classes under study, a supervised OPLS-DA was carried out. OPLS-DA technique separates the variation in *X* space (variables) into a predictive and an orthogonal part: the predictive part is correlated with *Y* space (classes) and the orthogonal one is uncorrelated to *Y*; therefore, the model interpretability is improved.

**Figure 3:**
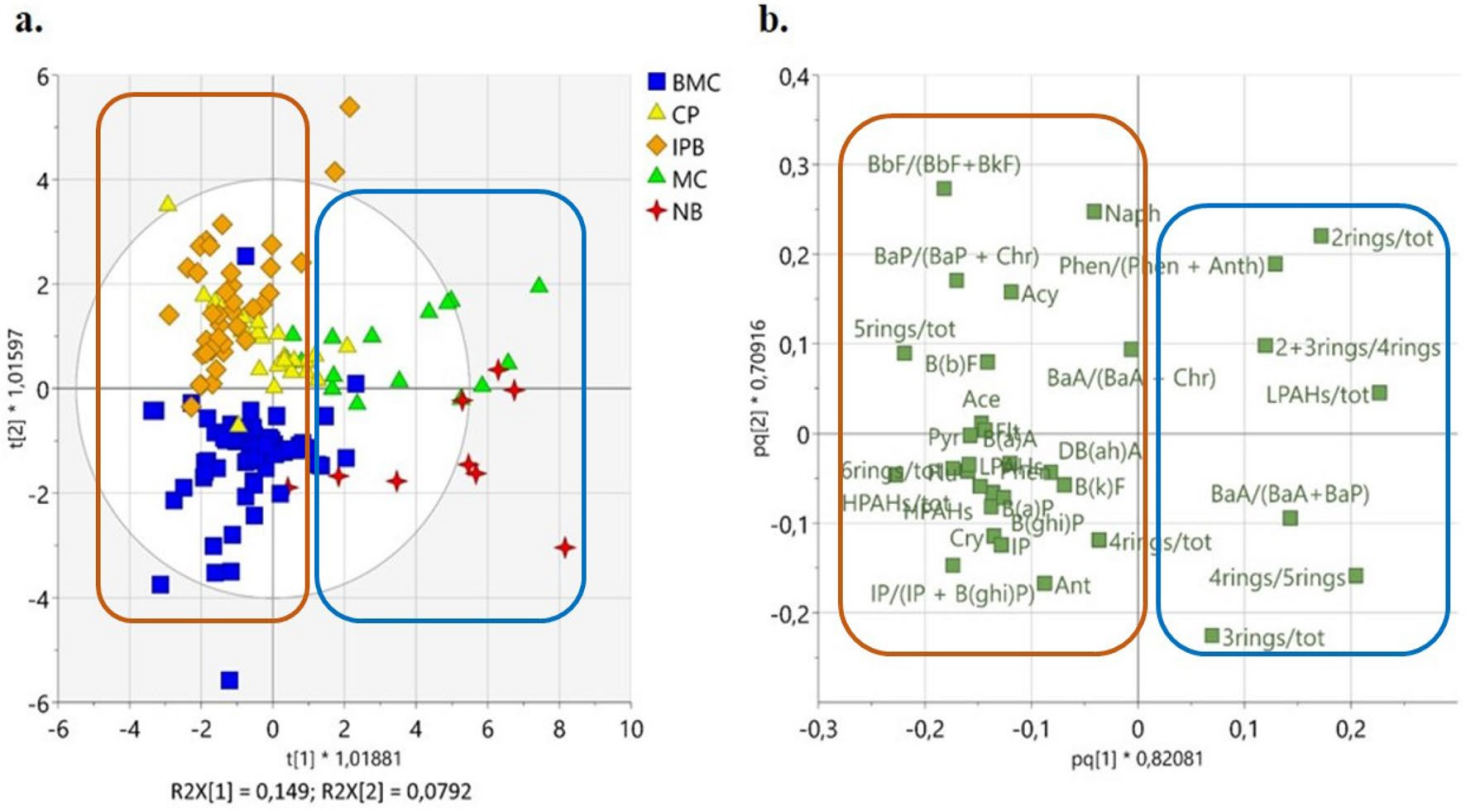
OPLS-DA applied to all classes (MC = Marine Coastal Transect; NB = Natural Bay; BMC = Mar Piccolo Basin; CP = Commercial Port; IPB = International relevance port): t1/t2 score plot (**a**) and p1/p2 loading plot (**b**), relative to the first two predictive components.

We obtained a 4 + 3 OPLS-DA model (4 predictive components and 3 orthogonal in *X*-space components) (Fig. 3), having *R*^2^ = 0.59 and *Q*^2^ = 0.52 (generally, values of (*R*^2^ − *Q*^2^) < 0.3 and *Q*^2^ > 0.5 are considered acceptable^54^ (Table S3). In the relative t1/t2 score plot (Fig. 3a), despite a slight heterogeneity existing within classes (BMC, CP, IPB, MC, and NB), a different PAHs pattern was distinguishable for each class. Two main groups of samples were highlighted (Fig. 3a). One group (encircled in blue), displayed around positive values of t1, included NB and MC classes (well separated from each other along t2). The other three classes (BMC, IPB, and CP) tended to group around negative values of t1 (encircled in orange); IPB and CP, partially overlapped, were characterized by positive value of t2, whereas BMC was displayed around negative values of t2.

The two groups reflected the different level of anthropogenic pressure: on one hand, NB and MC are influenced by a minor or negligible human and port activity; on the other hand, BMC, IPB, and CP are characterized by a relevant anthropic impact.

By observing the pq1/pq2 loading plot (Fig. 3b), it is apparent that NB and MC were characterized by a predominance of LPAHs with respect to IPB, BMC, and CP (Fig. S7a,b). This circumstance suggests that petroleum-related hydrocarbon inputs were the main PAHs sources in these basins^46^, probably due to oil loss from storage tanks used by fishery vessels anchored to the small shelters. In addition, MC well separated by NB samples: MC presented higher values of 2-rings/tot, while NB presented higher values of 3-rings/tot (Fig. S7c,d). The association of NB samples with BaA/(BaA + BaP) ratio and 4-rings/5-rings (besides the 3-rings/tot), suggests potential pyrogenic sources in the samples of this cluster (Fig. S7e,f). Indeed, the 4-rings/5-rings PAHs ratio indicates multiple pyrogenic sources and is commonly used to differentiate the tar sources from urban background^55,56^. The BaA/(BaA + BaP) and 4-rings/5-rings PAH ratios are also used coupled with PY/BaP ratio, especially for distinguishing terrigenous contribution into the aquatic system. According to De Luca et al.^6^, PY/BaP > 10 indicates petrogenic sources. In our case, analysing the BaA/(BaA + BaP) values obtained for NB samples, that ranged from 0.03 to 0.5, and comparing the Py/BaP values, that ranged from 1.0 to 53.39 with a mean of 12.58 (> 10), we infer a mixed (petrogenic and pyrolytic) origin of PAHs for NB^47,51^. These findings are coherent with the human-induced activities occurring in these two small shelters (SG and PL), with respect to the marine coastal area (MC) that was not influenced directly by any port activity.

The second main group, including the BMC, CP, and IPB classes, was further analysed by OPLS-DA.

#### OPLS-DA for ports under strong human-induced pressure

Aiming to easily identify a PAH pattern specific for each of the three classes (BMC, IPB, and CP), characterized by different kinds of anthropogenic activities having strong impact on the environment (Fig. 3), OPLS-DA was iterated by excluding NB and MC classes that showed a negligible anthropic pressure.

**Figure 4:**
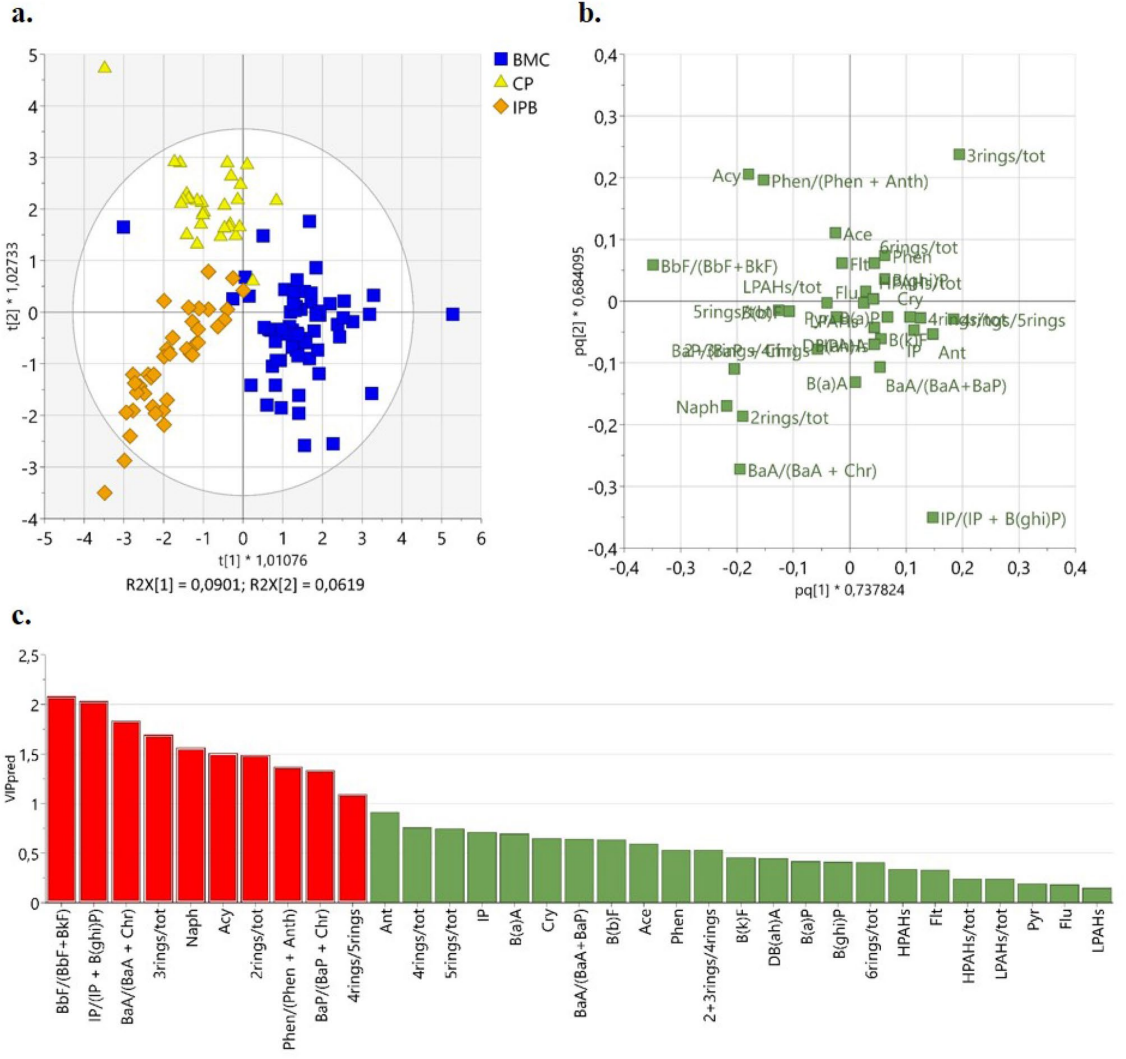
OPLS-DA applied on BMC (Mar Piccolo Basin), IPB (International relevance port), and CP (Commercial Port) samples: t1/t2 score plot (**a**) and pq1/pq2 loading plot (**b**), relative to the two predictive components; Predictive VIP plot (**c**) (variables with VIP value > 1 are marked in red).

A 2 + 2 OPLS-DA model was obtained (Fig. 4) showing *R*^2^ = 0. 68 and *Q*^2^ = 0.62, which are indicative of good descriptive and predictive power^54^. The detailed statistical parameters of the present model are reported in Table S4.

As shown in the t1/t2 score plot related to the 2 predictive components, the 3 classes BMC, IPB, and CP were clearly distinguished (Fig. 4a). BMC separated from IPB and CP along t1, whereas IPB and CP separated from each other along t2. It is apparent that the different kinds of activity carried out in these marine areas strongly governed the type on PAHs distribution. In order to identify the most discriminant variables for each class and to recognise the representative diagnostic ratios for each known source, the pq1/pq2 loading plot (Fig. 4b) and the predictive VIP plot were examined (Fig. 4c). The loading plot displays the weight of each variable on the selected principal components, while the predictive VIP plot displays the values of the Variable Importance in the Projection, a statistical parameter indicating the influence of each variable only on the predictive components (variables with VIP > 1 are considered more relevant on the classification model, albeit values up to 0.8 can be considered significant^54^ (Ericsson et al. 2013)).

The variables that mostly controlled the scattering of our observations were: the diagnostic ratios of IP/(IP + B(ghi)P), 4-rings/5-rings, BbF/(BbF + BkF), Phen/(Phen + Anth), BaA/(BaA + Chr), BaP/(BaP + Chr), 3-rings/tot, 2-rings/tot, along with the concentration values of some single congeners, such as Acy and Naph (marked in red in VIP plot).

**Figure 5:**
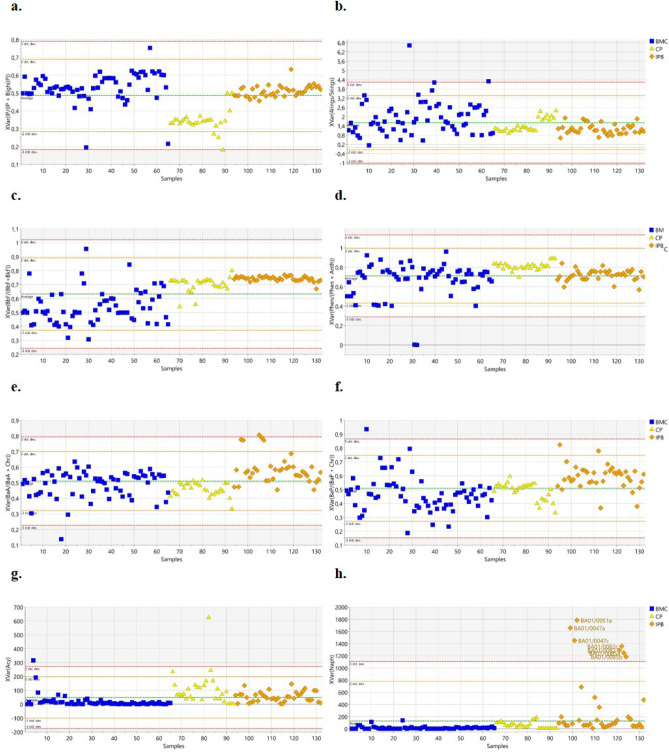
Statistic trend of the most significant variables (not scaled values) in BMC (Mar Piccolo Basin), IPB (International relevance port) and CP (Commercial Port) classes: (**a**) IP/(IP + BghiP), (**b**) 4-rings/5-rings, (**c**) BbF/(BbF + BkF), (**d**) Phen/(Phen + Anth), (**e**) BaA/(BaA + Chr), (**f**) BaP/(BaP + Chr), (**g**) Acenaphthylene (Acy), and (**h**) Naphtalene (Naph).

Figure 5 displays the original (not scaled) values of each variable marked in VIP graph. Among the most discriminant variables, the IP/(IP + B(ghi)P) ratio highly correlated with BMC, discriminating this cluster from the other two (IPB and CP). Indeed, the values of IP/(IP + B(ghi)P) resulted higher in BMC with respect to CP and, albeit to a less extent, in IPB (Fig. 5a), that, in turn, displayed intermediate values of this isomeric ratio. The BMC cluster resulted also associated with 4-rings/5-rings isomeric ratio (Fig. 5b).

High values of BbF/(BbF + BkF) and Phen/(Phen + Anth) isomeric ratios characterized the commercial ports (CP, Fig. 5c,d), while BaA/(BaA + Chr) ratio discriminated the IPB cluster (Fig. 5e).

The value of IP/(IP + B(ghi)P) ratio has already been used to differentiate between industrial coal combustions (high values) from combustion related to motor vehicle exhaust sources (low values)^8,19,20,51,57^. This is in line with our results: the proximity of the refinery and steel factory to the First bay of Mar Piccolo (BMC) where the BMC samples were collected^22,58,59^, can also explain the industrial pyrogenic PAH fingerprint in this area. The deposition of airborne particulate and combustion soot washed out by rain-off and transferred into the basin sediments could be the principal PAHs origin within BMC^49,50^. This hypothesis is also validated by the values of the IP/(IP + B(ghi)P) ranging from 0.2 to 0.7 (Table S2) that, according to Dvorská et al.^51^ (2011), are indicative of industrial sources (cement, coke, asphalt)..

**Figure 6:**
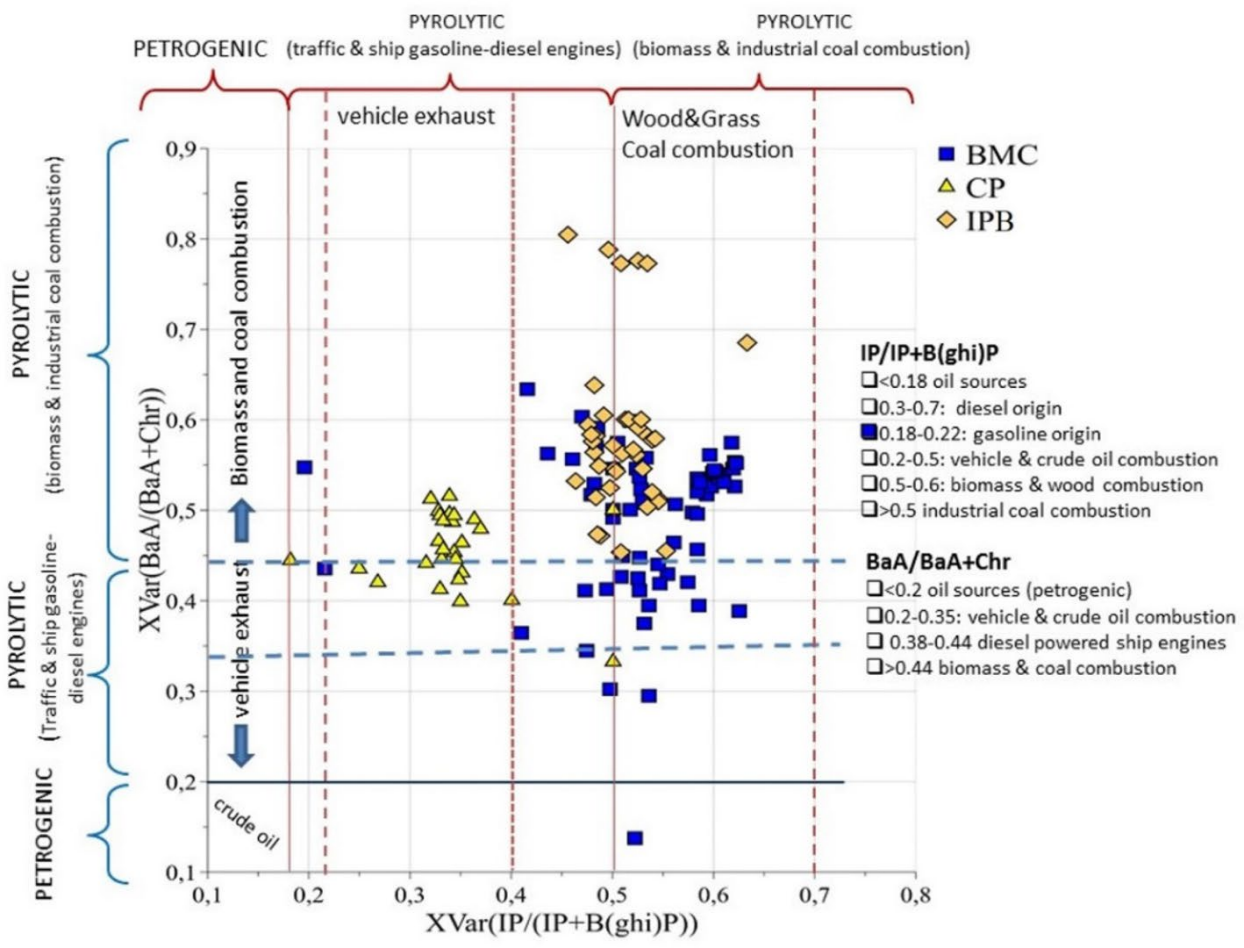
2D-plot displaying BaA/(BaA + Chr) versus IP/(IP + BghiP) isomeric ratios: intervals of values useful to attribute the PAH sources are identified.

Intermediate values of IP/(IP + B(ghi)P) were registered for IPB cluster (Fig. 5a) thus confirming an industrial PAHs fingerprint of the Port of Bari (IPB), albeit to a lesser extent with respect to the BMC. IPB samples resulted also associated with high values of the BaA/(BaA + Chr) and BaP/(BaP + Chr) isomeric ratios (Fig. 5e,f). Given that BaA is preferentially produced with respect to Chr during the combustion of fossil fuel and biomass^51^, the registered BaA/(BaA + Chr) and BaP/(BaP + Chr) ratios suggest mixed PAH sources from ship fuel combustions (probably by marine diesel engines used by ships and vessels) as well as from traffic exhaust^9,19,57,60,61^. However, such a conclusion holds rigorously when BaA and Chr are determined as soon as they are emitted. In fact, BaA can convert to Chr during degradation^6^ and this process is accelerated by organic matter content onto sediments. Thus, the use of BaA/(BaA + Chr) ratio to discriminate the type of combustions can lead to questionable results, because low values of BaA might derive from degradation processes. Therefore, we used the BaA/(BaA + Chr) ratio in conjunction with the IP/(IP + B(ghi)P) ratio to confirm the mixed sources in IPB samples^62^. The graph reporting the two paired diagnostic ratios IP/(IP + B(ghi)P) *vs* BaA/(BaA + Chr) is shown in Fig. 6. IPB samples have a PAHs pattern dominated by both vehicle/ships exhaust sources and coal combustions. Indeed, Port of Bari (IPB) is a multipurpose international hub with very heavy traffic emissions, from both sea and land area.

High values of B(b)F/(B(b)F + B(k)F) (Fig. 5c) logged in CP ports might be ascribed to urban traffic pollution. Lakhani et al.^9^ found that high values of B(b)F/B(k)F (> 1.2) in association with high concentrations of Flu and Py are originated by heavy-duty diesel vehicles. In our case, the average value of the B(b)F/B(k)F ratio is 2.5 (Table S2) and the relative % abundances of Flu and Py in CP reached the remarkable values of 16% and 14%, respectively (Table S1), confirming the influence of heavy-duty diesel vehicles to PAHs pattern in these areas.

High concentration of Acy found in commercial ports (CP) and, to less extent, in IPB (Fig. 5g) could be due to creosote used for vessels preservation, whose main constituents are naphthalene and acenaphthylene^63^. However, given the proximity of the commercial ports to the urban agglomerate, Acy might derive also from other commercial products like coal tar, coal tar pitch, creosote, bitumen, and asphalt^62^.

High concentration of Naph found in Port of Bari samples (IPB) and, to a less extent, in CP ports (Fig. 5h), with abnormal values logged in quays subjected to heavy passenger ships traffic (samples flagged with names in Fig. 5h), supported the hypothesis of pyrolytic PAH fingerprint related to traffic ships emissions^60^.

Finally, the predominance of 3-ringed PAHs in the sediments of CP (and in BMC as well) (Fig. S7d) suggested an effect of heavy traffic by both land and sea in these areas^64^.

#### OPLS-DA model validation

The 2+2 OPLS-DA model discussed above (see “OPLS-DA for ports under strong human-induced pressure” section) allowed us to pinpoint the PAHs isomeric ratios characteristic for each pollution source. It could be used not only as a descriptive model of the PAHs contamination in a specific area, but also as a tool to predict PAHs sources on the sole bases of specific PAH fingerprint. To test its robustness as a predictive model, a validation procedure was necessary. To this purpose, the goodness of the present OPLS-DA model was firstly cross-validated by means of the Receiver Operating Characteristic (ROC) plot (Fig. S8) and the misclassification table (Table 1), by using the work-set as the prediction-set.

**Table 1:**
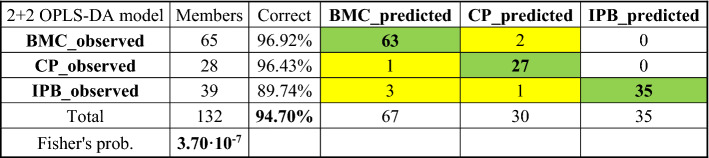
Misclassification table obtained for the OPLS-DA model, built on the BMC, IPB, and CP classes, by using the work-set itself as the prediction-set.

The ROC plot reports the ROC curves created by plotting the true positive rate (TPR) versus the false positive rate (FPR = 1 − TNR), based on the values of YPredPS (predicted *Y* values); the area under the curve (AUC) is a quantitative measure of the classification success, ranging between 0.5 (bad classification) and 1.0 (perfect classification)^54^. Excellent values of AUC were obtained for all of the three classes: specifically, 0.983 for BMC, 0.992 for CP, and 0.983 for IPB. Therefore, 94.7% of the observations was correctly classified, with a Fisher's probability (*p, *i.e. the probability of results occurring by chance, satisfied when *p* < 0.05 for 95% confidence) equal to 3.7·10^−7^ (Table 1). The class attribution was based on the major value of *Y*predPS, i.e. the predicted values for the variable *Y* (here, the class), computed for each observation (see the classification list in Table S5).

Additionally, a traditional univariate method was also applied to confirm the results of OPLS-DA model built for BMC, CP, and IPB. The non-parametric Kruskal–Wallis test was performed considering one at a time the most significant variables, indicated by VIP extracted for the three classes. The results confirmed that all the selected variables significantly discriminated between the three classes, validating thus the OPLS_DA outcomes. The details are reported in Appendix C of SI.

To further validate the current OPLS-DA model as a potential predictive tool for unknown samples, an external validation was also carried out. To this purpose, data from two research works have been exploited (Table S6), namely a work on an industrial port in the Southern Kaohsiung Harbor of Taiwan^65^ and a work on three Mediterranean ports: Cagliari (Italy), El Kantaoui (Tunisia), and Heraklion (Greece)^66^^.^

The validation set was composed by 50 samples: 14 samples (S1–S14) collected in the Southern Kaohsiung Harbor, a port extensively polluted by industrial wastewater discharges (1C1–3C5); 9 samples from El Kantaoui port, a quite recent small artificial marina (0.04 km^2^), located on the Eastern Tunisian coast within an international touristic center and surrounded by a small permanent population (1Ea–3Ec); 12 samples from Heraklion, an intermediate size port (0.87 km^2^), the main harbor of Crete and one of the most important ports in the Eastern Mediterranean, with an intense touristic and transport traffic (1H1–3H5)^66^.

Table 2 displays the values of *Y*predPS computed for each observation belonging to this external test set and the class attributed according to the major value of *Y*predPS (highlitghed in pink). The model attributed the samples from the Southern Kaohsiung Harbor of Taiwan, S1–S14, to BMC class, even if high values of *Y*predPS for IPB class were computed too. The results are coherent with the findings of the authors of the original publication, that described the industrial port area of Southern Kaohsiung harbor as affected by PAH pattern originated by oil/coal burning^65^. The harbour area is located near a highly industrialized area with features similar to those of the BMC (Basin of Mar Piccolo in Taranto city): the proximity of the China Steel Plan, the discharge of the Salt River that crosses the whole industrial discrict of Siaogang and the proximity of Talin Power Plan, justify the similarities of the PAHs pattern revelaed in this area with the BCM area. On the other hand, samples from the ports of Cagliari (1C1–3C5) were prevalently classified as ports with international relevance, thus with a considerable PAHs content (IPB). Also in this case, the results are consistent with the findings of the authors^66^. Port of Cagliari is a harbor area similar to that of Port of Bari (representative of IPB class). In line with Vitali’s results, Port of Cagliari resulted to be characterized by a PAHs pattern consistent with “a PAH origin from combustion of biomasses and coal”^66^. Attribution to IPB ports was obtained by our model also for samples collected in H1 and H3 sites of Heraklion ports. In fact, these sites are areas dedicated to passenger ships (H1) or leisure and fishing boats (H3), in full compliance with the land use purposes.

**Table 2:**
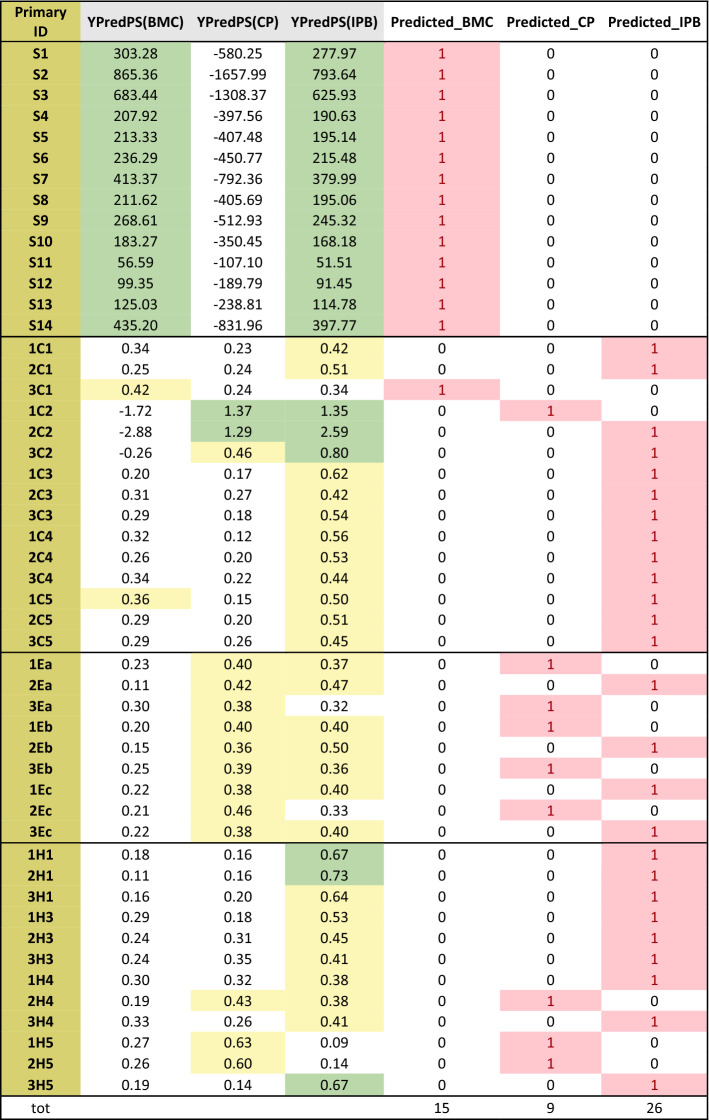
Prediction test on the external validation set.

Observations are colored based on YpredPS values: i. < 0.35 white (do not belong to the class); ii. 0.35–0.65 yellow (borderline); iii. > 0.65 green (belong to the class). The class attribution is based on the major value of YpredPS (pink).

As to samples from El Kantaoui, five of them were attributed to CP (like TM and MP) and four to IPB (like Port of Bari), showing a mixed PAH origin caused mainly by the incomplete combustion of fuels (i.e., gasoline, diesel, lubricating oils)^66^, as expected for a small port with a moderate marine traffic.

Interestingly, our OPLS-DA model attributed an unequivocal industrial fingerprint only to samples of Southern Kaohsiung Harbor of Taiwan, which were clearly differentiated from all other samples.

Finally, the attribution of El Kantaoui and Heraklion ports to CP was somehow equivocal, with *Y*predPS ranging from 0.35 to 0.65 (yellow highlights in Table 2), as noted by Vitali et al.^66^. We suppose that, despite the rather limited number of available observations per class and the strong differences of the test-observation selected, the present study could represent a good pilot study for constructing a robust model, aimed at PAHs pattern identification as well as prediction of pollution source attribution.

## Conclusions

In this work, the attribution of PAHs pollution sources in marine sediments was performed by submitting diagnostic PAH isomeric ratios to multivariate statistical analysis. Five areas subjected to different environmental pressures were investigated. The obtained OPLS-DA model allowed an accurate pollution source attribution, and consequently a reliable hazard degree assessment. The model enabled also to select the PAH diagnostic ratio useful for describing the predominant pollution source in marine sediments that are subjected to different human-induced pressures. The predictive capacity of the OPLS-DA model was validated exploiting an external dataset consisting of 50 samples from 4 port areas located in different continents.

The classification model set up here seems promising as starting point for future attempts implementation of source attribution statistical models based on PAHs patterns and PAH ratios.

Increasing the number of the training samples and thus, the representativeness of the pattern activities occurring within port areas, could provide a more robust and informative statistical model.

## Supplementary Information

Supplementary Information 1.

Supplementary Table S1.

Supplementary Table S2.

Supplementary Table S3.

Supplementary Table S4.

Supplementary Table S5.

Supplementary Table S6.

## Data Availability

All data generated or analyzed during this study are included in this published article [and its supplementary information files].
