# Supplementary material for: Improved identification of pollution source attribution by using PAH ratios combined with multivariate statistics
Source: Sci Rep. 2022 Nov 11;12:19298. doi: 10.1038/s41598-022-23966-4 (PMC9652473; doi:10.1038/s41598-022-23966-4)
Supplement: Supplementary file 1 — Supplementary Information 1. [file 41598_2022_23966_MOESM1_ESM.docx]

**SUPPLEMENTARY INFORMATION**

**Improved Identification of Pollution Source Attribution by using PAH Ratios Combined with Multivariate Statistics**

Matilda Mali*, Rosa Ragone, Maria Michela Dell’Anna, Giuseppe Romanazzi, Leonardo Damiani, Piero Mastrorilli

DICATECh, Politecnico di Bari, via Orabona, 4 I-70125 Bari, Italy

^*^Corresponding author e-mail address: [matilda.mali@poliba.it](mailto:matilda.mali@poliba.it) (M. Mali); <tel:+39> 080 5963666; fax: +39 080 5963414

**APPENDIX A**

Details on specific study areas selected for the present study:

Mar Piccolo Basin

1. The first area is a shallow marine basin in the southern part of Apulia Region, on the Ionian coastal side (Figure S1). The basin, named Mar Piccolo, is subjected to a sever industrial pressure and falls within a wider anthropized catchment, declared at high environmental risk from the National Government (Sollecito et al. 2019). The basin is constituted by two inlets connected with the Ionian Sea through two channels, one artificial (Navigable Channel) and one natural (Port Napoli Channel). The most important industrial activities are located close to the First inlet from which were selected the sample site for the present work. Among them, need to be mentioned one of the most important European steel factory that is still active in the area since 1960; the ENI Refinery and GPL Plant, an abandoned shipyard (built at the beginning of the twentieth century, 1889) and a very large Military Navy port located on the southern part of the first inlet. The industrialization of the area has completely distorted the natural ecosystem with the construction of piers, docks, tombstones, and dry docks, subjecting the seabed to different dredging activities and the overbuilding of kilometers and kilometers of shores with important impact on the land-sea marine track. Furthermore, intensive mussel culture and fishery facilities are still active in the first bay and affected seriously the quality of sediments and water column as documented by different studies [1][2][3][4].


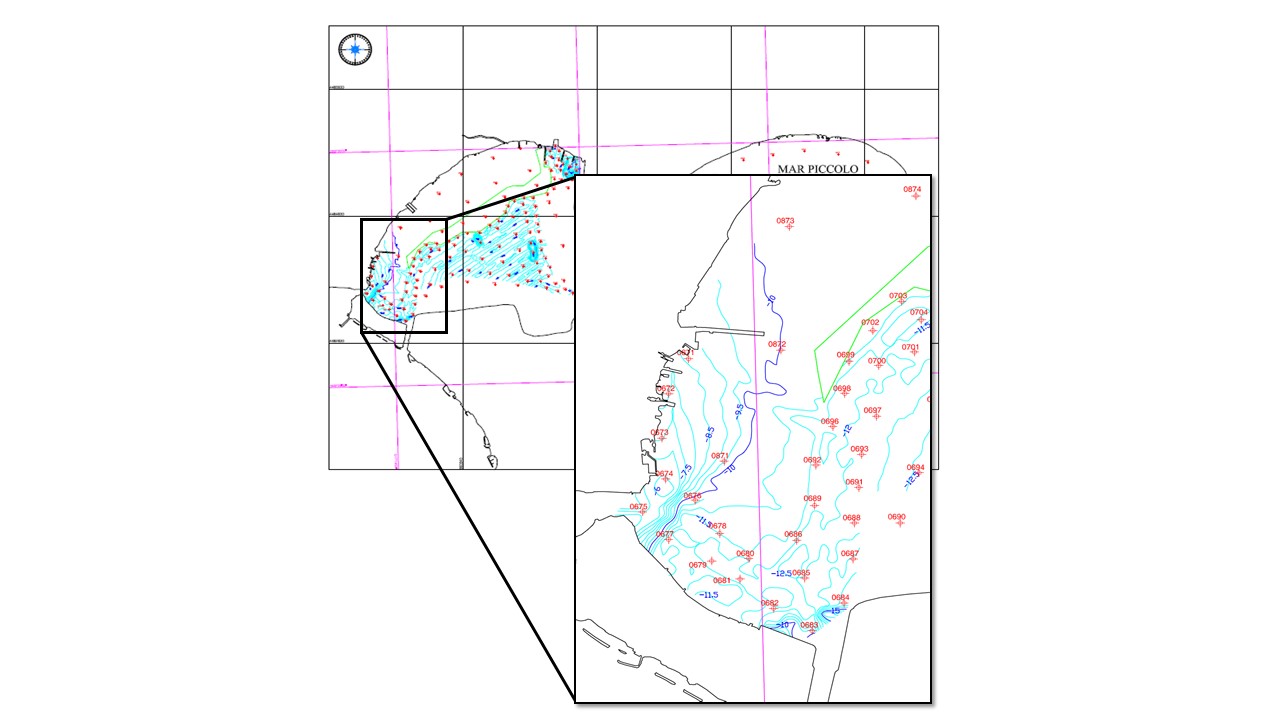


**Figure S1.** Mar Piccolo basin and the sites selected for the present study. The map was obtained using international WGS84 ellipsoid, UTM kilometric grid Fuso 33, (2010), modified using Power Point Software, License of the Polytechnic University of Bari.

1. The second selected study area is **the Port of Bari**, one of the most important ports of south-east Adriatic coast with an international relevance, near the urban area of Bari (Figure S2). The port has ancient origins. It is assumed to have been active since the invasion of the Apulian territory by the Iapyges. Currently it is a multipurpose stop-over equipped with docks for handling a range of goods and freight. Water depth varies from 6.9 to 14.9 m, allowing the movement of large ships and vessels. Several quays for various types of commercial and industrial traffic (solid and liquid bulk, containers, packaged goods, steel products, forestry products) (Container Terminal) as well as Ro-Ro docks for ferries (Ro-Ro terminal) and cruise ship platform (Cruise Terminal) and Passenger Terminal are daily used (Figure S2). High level of port activities has seriously impacted the quality of port aquatorium and therefore the quality of harbor sediments, as documented by [5][6][7]


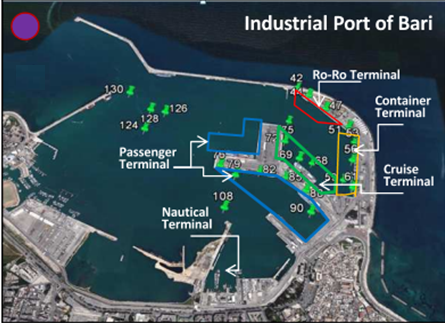


**Figure S2.** Port of Bari and selected sites for the present study. The map was obtained through Google Earth Software (https://www.google.it/intl/it/earth/, Data SIO, NOAA, U.S.Navy, NGA, GEBCO © 2015 Google) and further modified with Power Point Software—License to Polytechnic University of Bari (Italy).

1. The third study area selected is constituted by two ports: **Port of Monopoli (MP**) (Figure S3a) that is classified as commercial port, and **Torre a Mare port (TM)** (Figure S3b) mainly dealing with commercial and touristic activities. Port of Monopoli plays an important role in the Apulian territory, considered as a transit stopover and liaison between the vast hinterland and the sea. It is divided into two parts: the oldest port including three bays (the “Old Port” Bay, “The batteries" bay and "Fontanelle" bay) dedicated to mooring (Figure S3a), while on the recently developed part, is located the "Curatori" bay dedicated to shipyards and spaces for boat maintenance. The surface of the water body extends for approximately 140,000 m^2^. Field bathymetric surveys and studies showed an almost constant depth at the center of the harbor, approximately of 9 m [7][8]. Despite its modest extension, it is a commercial port with important touristic and recreational boating and activities that impact the quality of the port area, especially during summer, with the increasing of the fishery and yachting activities. On the other hand, the Port of Torre a Mare (TM) (Figure S3b), located between port of Bari and Port of Monopoli, is a commercial port having a more touristic vocation. Different commercial activities befall in the port such as fishery, bathing and yachting as well as maintenance and repair activities that impact the quality of the harbor basins. In summer, with the increasing of the fishery and yachting activities due to seasonal tourism demand, the human pressure results to be more consistent. It is supposed that the worsening of the quality of the coastal area might be attributable to tourism firms operating alongside the coast due to their unauthorized discharges. A previous hydrodynamic investigation on the port basin embedding the outer coastal area of the Torre a Mare [6], revealed that the TM port environmental situation is heavily influenced by what occurs outside the port area. The current regime favors the transportation of sediment associated contaminants towards the inner port area, where they accumulate in the stagnation zone in the oldest bays of the port [8][9].


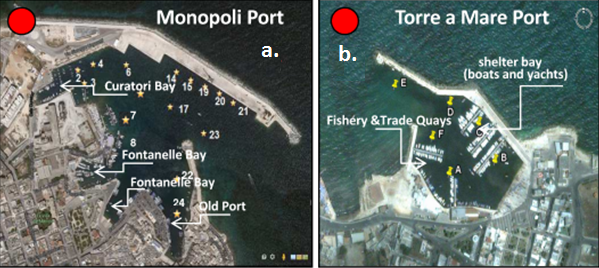


**Figure S3.** Port of Monopoli (a) Port of Torre a Mare (b) with the indication of sampling sites selected for the study. The map was obtained through Google Earth Software (<https://www.google.it/intl/it/earth/>, Data SIO, NOAA, U.S.Navy, NGA, GEBCO © 2015 Google) and further modified with Power Point Software—License to Polytechnic University of Bari (Italy).

1. The fourth selected area is constituted by two small ports with no record of past commercial activity: the shelter of Palese (PL) equipped with very limited port facilities and San Giorgio Bay (SG), a natural bay used mainly by the local fishermen as favorite anchorage for pleasure and fishing boats (Figure S4a.b). Both shelters are considered natural environment, thus not or slightly influenced by human‐induced pressures [7][10].


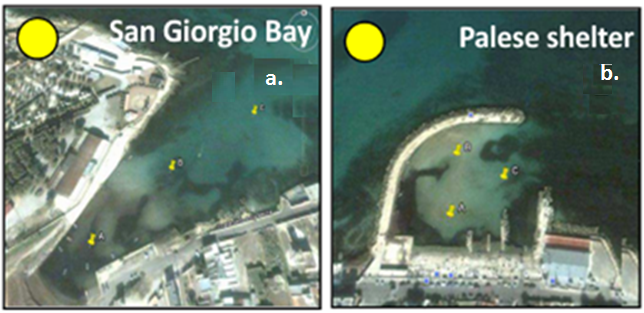


**Figure S4.** San Giorgio Bay (a) and Palese Shelter (b) with the indication of the sampling sites selected for the present study. The map was obtained through Google Earth Software (<https://www.google.it/intl/it/earth/>, Data SIO, NOAA, U.S.Navy, NGA, GEBCO © 2015 Google) and further modified with Power Point Software—License to Polytechnic University of Bari (Italy).

1. Aiming to complete the different pattern areas, nine coastal marine sites were included in the present study (Figure S5). These sites are situated at 200 m and 500 m from the coast (marine transect) and are located between the Ofanto River estuary (MC FO1 and MC FO2) and the southern part of Monopoli (MC MO1 and MC MO2). Two samples were selected near the Bari coastal area (MC BA1 and MC BA2) and other two samples were collected in the coastal track in front of the two small ports (PL and SG). These areas are free from human pressure, even if very close to the coastline. However, they are subjected to marine currents, and thus considered not directly influenced by contamination and representative of almost pristine condition.


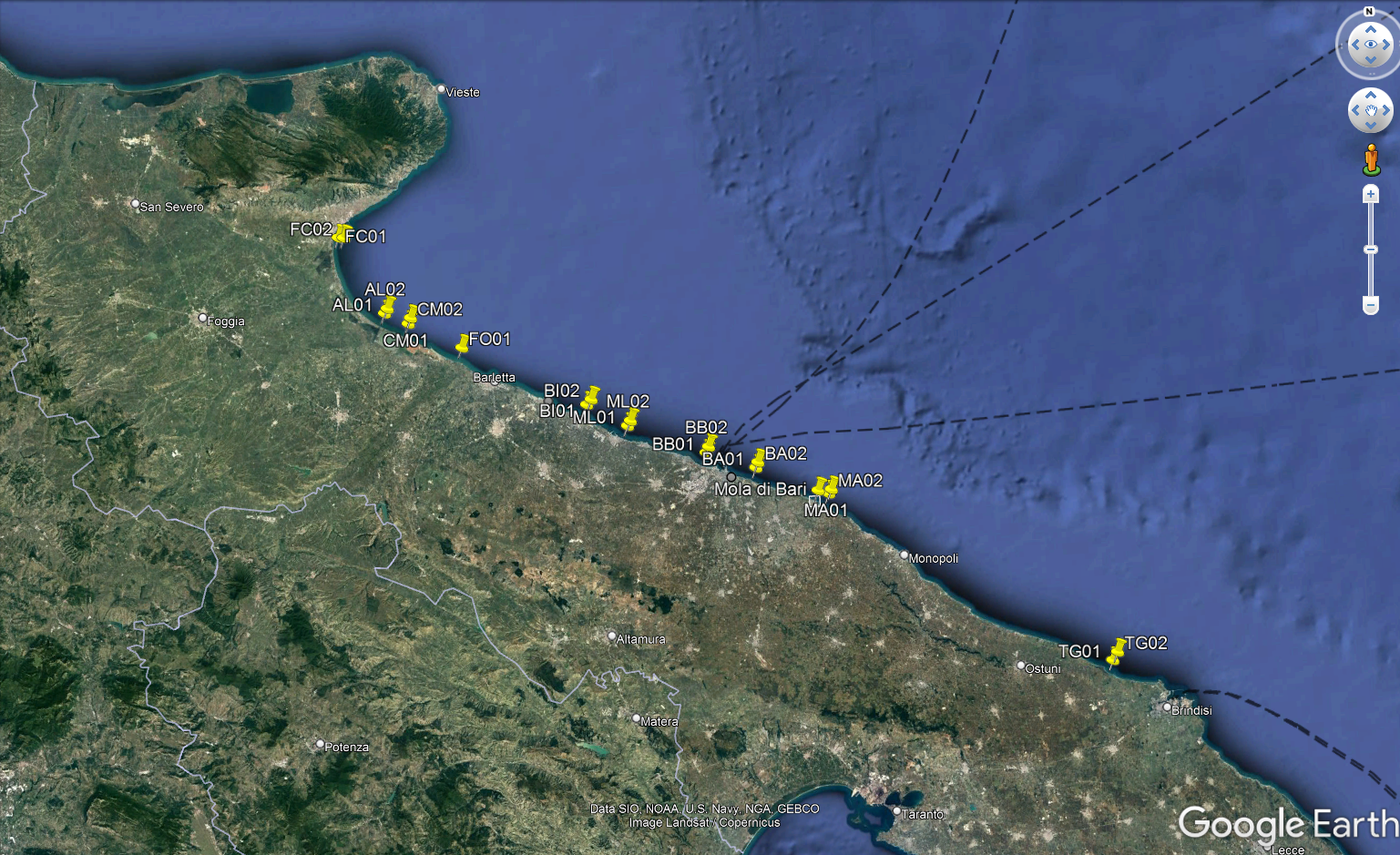


**Figure. S5.** The track of marine coastal area selected for the present study. The map was obtained through Google Earth Software (<https://www.google.it/intl/it/earth/>, Data SIO, NOAA, U.S.Navy, NGA, GEBCO © 2015 Google) and further modified with Power Point Software—License to Polytechnic University of Bari (Italy).

**References for S.I. Appendix A**

1. ISPRA (Istituto Superiore per la Protezione e la Ricerca Ambientale). Elaboration and evaluation of the results of the characterization for the purpose of identifying the appropriate interventions for the safety and remediation of the Site of National Interest of Taranto—Mar Grande II lot and Mar Piccolo. CII-El-PU-TA-Mar Grande II Lotto e Mar Piccolo-01.06 Agosto 2010.
2. Cardellicchio, N. et al. Organic pollutants (PAHs, PCBs) in sediments from the Mar Piccolo in Taranto (Ionian Sea, Southern Italy). Mar. Pollut. Bull. 55, 451-458 (2007) <https://doi.org/10.1016/j.marpolbul.2007.09.007>
3. Di Leo, A. *et al*. Monitoring of PCDD/Fs and dioxin-like PCBs and seasonal variations in mussels from the Mar Grande and the Mar Piccolo of Taranto (Ionian Sea, Southern Italy). *Environ. Sci. Pollut. Res*. **21 (23),** 13196-13207 (2014).
4. Mali, M., Dell’Anna, M. M., Notarnicola, M., Damiani, L. & Mastrorilli, P. Combining chemometric tools for assessing hazard sources and factors acting simultaneously in contaminated areas. Case study: “Mar Piccolo” Taranto (South Italy). *Chemosphere* **184**, (2017b).
5. Mali, M. *et al.* Are conventional statistical techniques exhaustive for defining metal background concentrations in harbour sediments? A case study: The Coastal Area of Bari (Southeast Italy). *Chemosphere* **138**, (2015).
6. Mali, M., Malcangio, D., Dell’ Anna, M. M., Damiani, L. & Mastrorilli, P. Influence of hydrodynamic features in the transport and fate of hazard contaminants within touristic ports. Case study: Torre a Mare (Italy). *Heliyon* **4**, (2018).
7. Mali, M. *et al.* Identification of hot spots within harbour sediments through a new cumulative hazard index. Case study: Port of Bari, Italy. *Ecological Indicators* **60**, (2016).
8. Mali, M., Dell’Anna, M. M., Mastrorilli, P., Damiani, L. & Piccinni, A. F. Assessment and source identification of pollution risk for touristic ports: Heavy metals and polycyclic aromatic hydrocarbons in sediments of 4 marinas of the Apulia region (Italy). *Marine Pollution Bulletin* **114**, (2017).
9. Malcangio, D., Melena, A., Damiani, L., Mali, M. & Saponieri, A. Numerical study of water quality improvement in a port through a forced mixing system. *WIT Transactions on Ecology and the Environment* **220**, (2017).
10. Mali, M. *et al.* Are conventional statistical techniques exhaustive for defining metal background concentrations in harbour sediments? A case study: The Coastal Area of Bari (Southeast Italy). *Chemosphere* **138**, (2015).

**APPENDIX B**

Cross Validation Procedure PCA-OPLS_DA:

### Cross validation for PCA

1. Parts of the X data are kept out of model development. SIMCA uses the approach of Krzanowski where in two sub rounds, data are first kept out observation-wise (row-wise) to get a set of loading vectors, and second data are kept out variable-wise (column-wise) to get a set of score vectors.
2. The kept-out parts are then predicted by the model.
3. The predictions of the kept-out parts are compared with the actual values.
4. 1-3 is repeated until all parts have been kept out once and only once.

The prediction error sum of squares (PRESS) is the squared differences between observed and predicted values for the data kept out of the model fitting. The prediction of the (i, k) element in scaled and centered form is the ith score value multiplied by the kth loading value, where both have been estimated in a CV round when this element was kept out.

This procedure is repeated several times until every data element has been kept out once and only once. The final PRESS then has contributions from all data.

For each component consecutively, SIMCA computes the overall PRESS/SS, where SS is the residual sum of squares of the previous component. A special (proprietary) PC estimation is used in the CV rounds to minimize the tendency for the present component to partly rotate into later components.

### Cross validation for OPLS

1. Rows of the X/Y-data are kept out of model development
2. The kept out parts are then predicted by the model.
3. The predictions of the kept out parts are compared with the actual values.
4. 1-3 is repeated until all parts have been kept out once and only once.

The prediction error sum of squares (PRESS) is the squared differences between observed and predicted values for the Y-data kept out of the model fitting. The prediction of the (i, m) element in scaled and centered form is the i^th^ score value multiplied by the m^th^ loading value, where both have been estimated in a CV round when this element was kept out.

This procedure is repeated several times until every data element has been kept out once and only once. The final PRESS then has contribution from all data.

#### OPLS Specific

The OPLS validation is performed as follows:

1. The number of predictive or joint X/Y components is estimated; this number may be adjusted using rule R2 after calculating all predictive and orthogonal components.
2. The number of orthogonal components in X and Y are decided.

For these steps, rule R1 is used to determine the significance of the components.

For all included components, SIMCA computes the overall Q^2^ = (1-PRESS/SS), where SS is the sum of squares of Y. This type of cross validation is called full cross validation.

The cross validation for the Orthogonal in X(PCA) and the Orthogonal in Y(PCA) is performed as for the regular PCA above.

### Cross validation rules - Significant component

#### Rule 1: R1

A component is significant according to Rule 1 when Q^2^ > Limit

where

Limit = 0 for PLS models with more than 100 observations.

Limit = 0.05 for PLS models with 100 observations or less.

Limit = 0.01 for OPLS.

Limit depends on the number of components for PCA. The limit increases with subsequent components to account for the loss in degrees of freedom.

#### Rule 2: R2 for PCA

A component is significant according to **Rule 2 when**

Q^2^V> Limit for at least

- 20% of the x-variables when K > 25.
- sqrt(K)*log10(max (10, K-20)) when K < 25.

K = number of x-variables.

Q^2^V is Q^2^ for individual variables.

Provided the eigenvalue > 1.5 or K < 30.

#### Rule 2: R2 for OPLS

A component is significant according to **Rule 2 when**

Q^2^V> Limit for at least

- 20% of the y-variables when M > 25.
- sqrt(M) when M < 25.

M = number of y-variables.

Q^2^V is Q^2^ for individual variables.

#### Rule 3: U and R5

With PCA when a component is insignificant it is first labeled U (Undecided). If the next component is significant and has similar eigenvalues (tolerance of 5%) as the previous one, then both components together are considered significant. The U of the undecided component is changed to R5

**Figure S6**


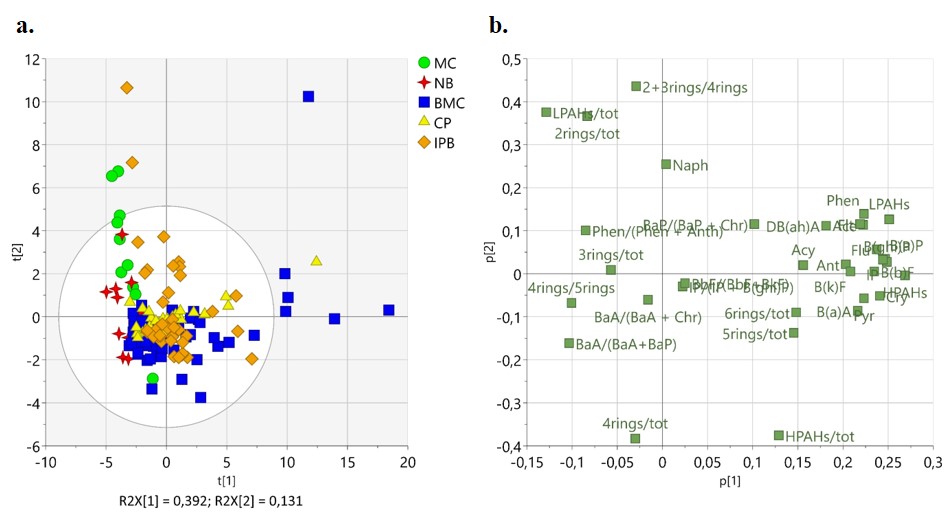


**Figure S6**. PC1 vs PC2 score plot of the PCA Model (a), PC1 vs PC2 loading plots of the PCA Model.

**Figure S7**


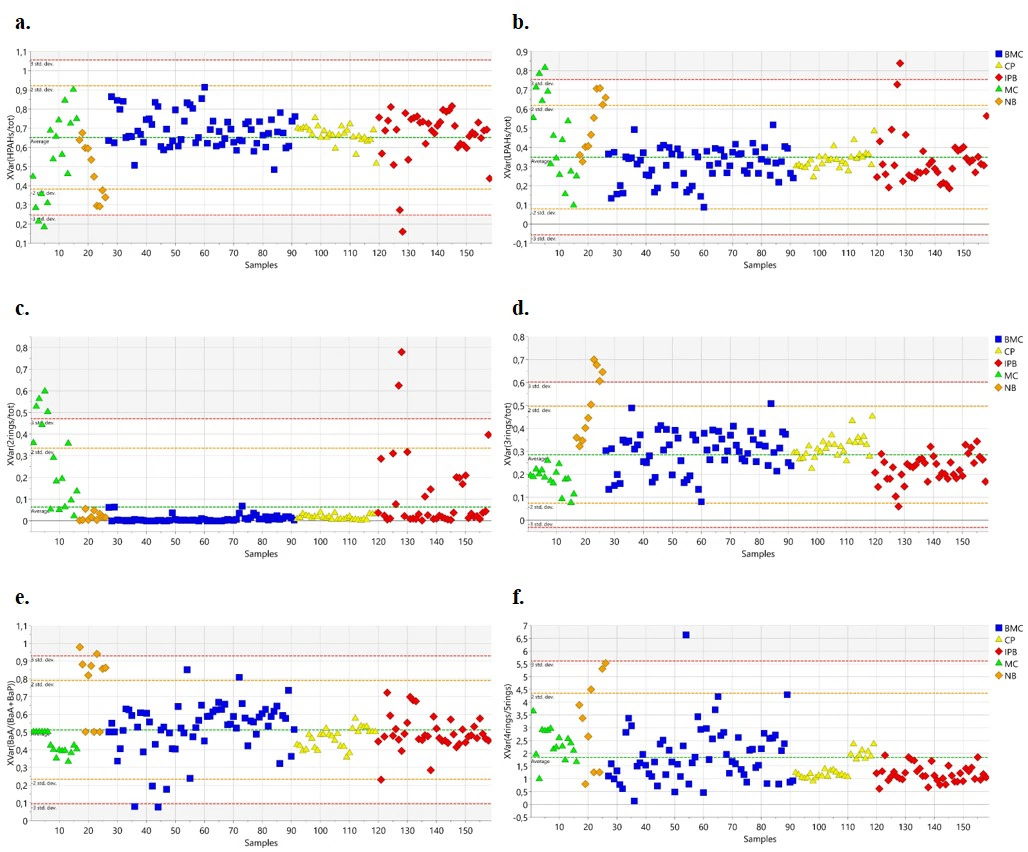


**Figure S7.** Trend of Diagnostic Molecular Ratios considered for the 5 classes (BMC, CP, IPB, MC and NB)

**Figure S8**


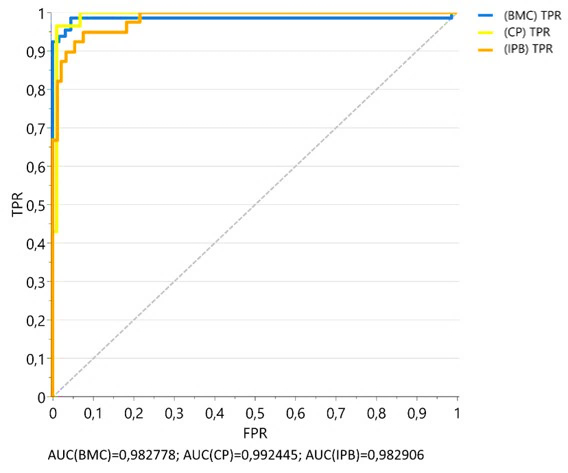


**Figure S8.** Receiver Operating Characteristic (ROC) plot for the model 2+2 OPLS_DA. the area under the curve (AUC) is a quantitative measure of the classification success, ranging between 0.5 (bad classification) and 1.0 (perfect classification).

**APPENDIX C**

A Kruskal-Wallis test was performed on the original values of the most significant variables (selected on the basis of VIP values) one at a time, by using an online calculator. We applied this non-parametric test because the variables selected were not normally distributed within each group (class), every group included false “outliers” that could not be removed, and the three groups had a different number of observations (n(BMC) = 65; n(CP) = 28; n(IPB) = 39). This test is based on the ranks of data and determines whether or not the groups of observations come from populations with the same median. Two hypotheses are set: the null hypothesis “H0: The samples come from populations with equal medians” against the alternative hypothesis “Ha: The samples come from populations with medians that are not all equal”. Given a fixed significance level and a number of degrees of freedom df = k–1 (k = number of classes), the rejection region of the null hypothesis is R = {χ2 : χ2 > χ2U }, where χ2 = test statistic H computed and χ2U = test statistic H tabulated.

The test statistic H, χ2, is computed as follows:

where N is the total number of observations, and Ri is the sum of ranks for group i, from a total of k group. When there are at least 5 observations per group, the test statistic H is approximated by a Chi-Square distribution with k – 1 degrees of freedom. In our case, k = 3, then df = 2, and N = 132 (65 + 28 + 39); we set the significance level α = 0.05.

Table S7 summarizes the tests computed for each of the 3 classes (BMC, CP, and IPB).

Table S7. In brief, the results obtained by applying the Kruskal-Wallis test on the most discriminant variables, selected on the basis of VIP values, for the three classes of BMC, CP, and IPB.

| **Variable** | **ΣR(BMC)** | **ΣR(CP)** | **ΣR(IPB)** | **df** | **χ2** | **χ2_U_** | **p** |
| --- | --- | --- | --- | --- | --- | --- | --- |
| BbF/(BbF + BkF) | 2496.5 | 2174.5 | 4107 | 2 | 77.594 | 5.991 | 0 |
| IP/(IP + B(ghi)P) | 7143 | 1765 | 3653 | 2 | 88.949 | 5.991 | 0 |
| BaA/(BaA + Chr) | 3965 | 1014.5 | 3798.5 | 2 | 44.326 | 5.991 | 0 |
| 3-rings/tot | 4919 | 2363.5 | 1495.5 | 2 | 31.011 | 5.991 | 0 |
| Naph | 2638 | 2103 | 4037 | 2 | 67.776 | 5.991 | 0 |
| Acy | 2753.5 | 2726 | 3298.5 | 2 | 52.822 | 5.991 | 0 |
| 2-rings/tot | 2939.5 | 2096 | 3742.5 | 2 | 44.588 | 5.991 | 0 |
| BaP/(BaP + Chr) | 3277.5 | 1680.5 | 3820 | 2 | 38.653 | 5.991 | 0 |
| Phen/(Phen + Anth) | 3526.5 | 2977.5 | 2274 | 2 | 38.829 | 5.991 | 0 |

where ΣR = the sum of ranks for each class; df = degree of freedom k–1 (where k = number of classes); χ2 = test statistic H computed; χ2U = the test statistic H tabulated for α = 0.05 and df = k – 1.

Since χ2 > χ2U and p-value < 0.05 for all the variables selected, the null hypothesis was rejected for all of them, that is the medians of the groups compared were not equal. Thus, we could safely claim that all the variables selected significantly discriminated between the three classes of BMC, CP, and IPB.
