# Supplementary material for: Improved identification of pollution source attribution by using PAH ratios combined with multivariate statistics
Source: Sci Rep. 2022 Nov 11;12:19298. doi: 10.1038/s41598-022-23966-4 (PMC9652473; doi:10.1038/s41598-022-23966-4)
Supplement: Supplementary file 2 — Supplementary Table S1. [file 41598_2022_23966_MOESM2_ESM.docx]

**Table S1.** Basic statistic data of 16 EPA PAHs concentration level and groups of PAHs (LPAHs, HPAHs, 2-ringed PAHs, 3-ringed PAHs, 4-ringed PAHs, 5-ringed PAHS and 6-ringed PAHs, and 𝛴16 PAHs), in the sediments of the selected areas

| **Areas** | **Statistic Data** | **Naph** | **Anth** | **Phen** | **Ace** | **Acy** | **Flu** | **Flt** | **Py** | **Chr** | **B(a)A** | **B(a)P** | **B(b)F** | **B(k)F** | **DBahA** | **B(ghi)P** | **IP** | **LPAHs** | **HPAHs** | **2rings** | **3rings** | **4rings** | **5rings** | **6rings** | **𝛴16PAHs** |
| --- | --- | --- | --- | --- | --- | --- | --- | --- | --- | --- | --- | --- | --- | --- | --- | --- | --- | --- | --- | --- | --- | --- | --- | --- | --- |
| **(MC)** | MIN | 11.74 | 0.50 | 2.55 | 0.50 | 0.50 | 0.50 | 0.50 | 0.50 | 0.50 | 0.50 | 0.50 | 0.50 | 0.50 | 0.50 | 0.50 | 0.50 | 17.31 | 5.00 | 11.74 | 4.55 | 2.00 | 2.00 | 1.00 | 23.32 |
|  | MAX | 55.00 | 20.00 | 65.00 | 5.00 | 11.00 | 9.00 | 98.50 | 88.50 | 69.30 | 45.80 | 62.00 | 39.00 | 34.40 | 8.60 | 41.50 | 33.40 | 125.00 | 521.00 | 55.00 | 101.00 | 302.10 | 144.00 | 74.90 | 577.50 |
|  | STD | 11.28 | 5.59 | 19.93 | 1.30 | 2.82 | 2.71 | 32.53 | 27.83 | 20.51 | 14.05 | 19.96 | 11.02 | 10.71 | 2.83 | 13.03 | 10.49 | 34.96 | 160.29 | 11.28 | 29.70 | 93.88 | 44.20 | 23.44 | 182.39 |
|  | Gmean | 19.09 | 1.79 | 10.91 | 0.89 | 1.21 | 1.94 | 10.02 | 8.83 | 5.23 | 3.18 | 4.21 | 2.62 | 3.17 | 1.43 | 5.40 | 3.85 | 40.23 | 49.79 | 19.09 | 17.58 | 27.70 | 12.12 | 9.30 | 101.78 |
|  | Mean | 21.08 | 4.06 | 18.21 | 1.23 | 2.06 | 2.84 | 25.65 | 21.98 | 14.36 | 9.86 | 14.49 | 7.51 | 8.03 | 2.50 | 11.76 | 8.95 | 49.46 | 125.08 | 21.08 | 28.39 | 71.85 | 32.53 | 20.70 | 174.54 |
|  | **% ab^[[1]](#footnote-1)^** | **12%** | **2%** | **10%** | **1%** | **1%** | **2%** | **15%** | **13%** | **8%** | **6%** | **8%** | **4%** | **5%** | **1%** | **7%** | **5%** | **28%** | **72%** | **12%** | **16%** | **41%** | **19%** | **12%** | **100%** |
| **(NB)** | MIN | 0.50 | 2.00 | 2.50 | 0.50 | 2.00 | 2.50 | 2.50 | 2.50 | 2.50 | 2.50 | 2.50 | 2.50 | 2.50 | 0.50 | 2.50 | 2.50 | 18.50 | 23.00 | 0.50 | 16.00 | 10.00 | 8.00 | 5.00 | 46.00 |
|  | MAX | 2.50 | 34.74 | 106.87 | 6.00 | 34.74 | 211.05 | 7.00 | 161.74 | 80.87 | 117.01 | 15.96 | 43.76 | 43.40 | 5.00 | 43.76 | 2.50 | 379.84 | 515.48 | 2.50 | 377.34 | 362.11 | 107.11 | 46.26 | 763.24 |
|  | STD | 0.63 | 10.64 | 29.81 | 2.49 | 10.64 | 85.07 | 1.55 | 58.96 | 31.81 | 44.17 | 5.63 | 17.44 | 16.81 | 2.26 | 17.44 | 0.00 | 121.29 | 188.90 | 0.63 | 121.47 | 133.80 | 39.75 | 17.44 | 286.36 |
|  | Gmean | 2.13 | 5.35 | 19.52 | 2.70 | 5.35 | 49.24 | 2.97 | 21.10 | 10.32 | 17.17 | 3.62 | 5.65 | 5.58 | 1.95 | 5.65 | 2.50 | 100.46 | 91.68 | 2.13 | 96.49 | 57.32 | 18.92 | 9.36 | 200.25 |
|  | Mean | 2.30 | 9.22 | 31.39 | 4.05 | 9.22 | 91.81 | 3.20 | 52.85 | 26.58 | 39.15 | 5.17 | 13.20 | 12.77 | 3.10 | 13.20 | 2.50 | 147.97 | 171.72 | 2.30 | 145.67 | 121.77 | 34.24 | 15.70 | 319.69 |
|  | **% ab** | **1%** | **3%** | **10%** | **1%** | **3%** | **29%** | **1%** | **17%** | **8%** | **12%** | **2%** | **4%** | **4%** | **1%** | **4%** | **1%** | **46%** | **54%** | **1%** | **46%** | **38%** | **11%** | **5%** | **100%** |
| **(BMC)** | MIN | 0.50 | 0.50 | 0.50 | 0.50 | 0.50 | 0.50 | 0.50 | 0.50 | 0.50 | 0.50 | 0.50 | 0.50 | 0.50 | 0.50 | 0.50 | 0.50 | 3.00 | 5.00 | 0.50 | 2.50 | 2.00 | 2.00 | 1.00 | 8.00 |
|  | MAX | 140.00 | 3650.00 | 4870.00 | 196.00 | 315.0 | 5010.0 | 407.0 | 4950.0 | 3970.0 | 4100.0 | 4510.0 | 3060.0 | 4420.0 | 2080.00 | 3410.0 | 3730.0 | 10618.3 | 29692.0 | 140.00 | 10504.3 | 13243.0 | 10979.0 | 7140.0 | 39702.40 |
|  | STD | 24.14 | 652.71 | 734.18 | 33.12 | 46.35 | 974.99 | 60.67 | 857.74 | 768.72 | 705.08 | 983.45 | 582.64 | 799.10 | 377.25 | 866.21 | 902.02 | 2124.02 | 5846.99 | 24.14 | 2112.11 | 2235.44 | 2342.11 | 1758.0 | 7720.21 |
|  | Gmean | 5.73 | 64.40 | 121.44 | 10.40 | 5.47 | 285.31 | 15.92 | 200.27 | 196.30 | 186.75 | 173.93 | 75.41 | 62.94 | 25.55 | 114.25 | 130.86 | 580.22 | 1355.62 | 5.73 | 562.64 | 637.50 | 389.77 | 248.96 | 1977.86 |
|  | Mean | 15.63 | 296.87 | 438.18 | 24.06 | 19.40 | 781.08 | 38.40 | 615.06 | 578.99 | 513.74 | 644.59 | 306.79 | 336.24 | 153.98 | 525.16 | 551.76 | 1575.23 | 4264.71 | 15.63 | 1559.59 | 1746.19 | 1441.61 | 1076.9 | 5839.94 |
|  | **% ab** | **0%** | **5%** | **8%** | **0%** | **0%** | **13%** | **1%** | **11%** | **10%** | **9%** | **11%** | **5%** | **6%** | **3%** | **9%** | **9%** | **27%** | **73%** | **0%** | **27%** | **30%** | **25%** | **18%** | **100%** |
| **(CP)** | MIN | 5.00 | 2.50 | 20.00 | 0.50 | 2.50 | 20.00 | 2.50 | 25.00 | 10.00 | 5.00 | 5.00 | 10.00 | 2.50 | 0.50 | 10.00 | 10.00 | 50.50 | 80.50 | 5.00 | 45.50 | 42.50 | 18.00 | 20.00 | 153.50 |
|  | MAX | 185.00 | 625.00 | 2350.00 | 252.00 | 625.0 | 3551.0 | 288.0 | 2976.0 | 1789.0 | 1444.0 | 2013.0 | 2381.0 | 859.00 | 214.00 | 2381.0 | 1258.0 | 7545.00 | 15603.0 | 185.00 | 7403.00 | 6497.00 | 5467.00 | 3639.0 | 23148.00 |
|  | STD | 52.06 | 120.08 | 481.81 | 49.83 | 120.0 | 785.31 | 55.76 | 666.69 | 396.69 | 304.31 | 446.82 | 539.58 | 194.83 | 53.94 | 539.58 | 284.52 | 1579.60 | 3464.46 | 52.06 | 1543.35 | 1416.26 | 1230.56 | 823.84 | 5035.12 |
|  | Gmean | 31.47 | 59.51 | 257.07 | 12.85 | 59.51 | 401.26 | 26.54 | 376.44 | 189.87 | 158.60 | 181.91 | 238.70 | 101.42 | 25.20 | 238.70 | 122.92 | 844.72 | 1691.09 | 31.47 | 802.70 | 756.23 | 552.25 | 364.52 | 2548.50 |
|  | Mean | 57.25 | 106.88 | 440.21 | 33.98 | 106.9 | 729.79 | 46.70 | 649.25 | 352.07 | 288.79 | 379.29 | 483.11 | 196.02 | 56.20 | 483.11 | 249.75 | 1474.98 | 3184.27 | 57.25 | 1417.73 | 1336.80 | 1114.61 | 732.86 | 4659.25 |
|  | **% abb** | **1%** | **2%** | **9%** | **1%** | **2%** | **16%** | **1%** | **14%** | **8%** | **6%** | **8%** | **10%** | **4%** | **1%** | **10%** | **5%** | **32%** | **68%** | **1%** | **30%** | **29%** | **24%** | **16%** | **100%** |
| **(IPB)** | Min | 8.15 | 9.38 | 25.08 | 0.50 | 2.21 | 54.97 | 5.97 | 58.65 | 22.66 | 29.36 | 45.26 | 53.13 | 16.80 | 16.39 | 35.24 | 38.68 | 240.77 | 366.41 | 8.15 | 100.25 | 118.84 | 144.55 | 73.92 | 691.06 |
|  | Max | 1781.71 | 426.62 | 1294.26 | 106.93 | 145.3 | 3015.4 | 191.3 | 4203.1 | 1160.8 | 2297.0 | 1748.7 | 2395.3 | 742.65 | 343.55 | 1045.6 | 1268.0 | 5029.21 | 14709.2 | 1781.7 | 4833.35 | 7642.20 | 5215.96 | 2150.2 | 18046.88 |
|  | STD | 527.89 | 99.58 | 278.77 | 22.23 | 36.84 | 617.93 | 36.28 | 780.68 | 267.56 | 496.59 | 375.73 | 514.91 | 159.84 | 79.88 | 224.00 | 267.17 | 1041.95 | 3015.61 | 527.89 | 995.89 | 1494.81 | 1123.42 | 485.09 | 3863.48 |
|  | Gmean | 143.98 | 77.69 | 201.21 | 17.65 | 37.89 | 479.11 | 36.30 | 499.70 | 209.66 | 300.68 | 308.10 | 389.04 | 134.69 | 86.93 | 230.98 | 241.80 | 1228.51 | 2499.59 | 143.98 | 838.24 | 1068.54 | 927.33 | 473.73 | 3890.36 |
|  | Media | 366.72 | 112.87 | 294.62 | 24.96 | 54.06 | 696.33 | 46.30 | 755.35 | 304.12 | 489.30 | 427.95 | 558.87 | 187.97 | 113.91 | 308.91 | 329.63 | 1549.56 | 3522.31 | 366.72 | 1182.84 | 1595.06 | 1288.70 | 638.54 | 5071.87 |
|  | **% ab** | **7%** | **2%** | **6%** | **0%** | **1%** | **14%** | **1%** | **15%** | **6%** | **10%** | **8%** | **11%** | **4%** | **2%** | **6%** | **6%** | **31%** | **69%** | **7%** | **23%** | **31%** | **25%** | **13%** | **100%** |

1. * ab = relative abundance of the given PAH congener to the 𝛴16 EPA PAHs [↑](#footnote-ref-1)
