# Supplementary material for: Improved identification of pollution source attribution by using PAH ratios combined with multivariate statistics
Source: Sci Rep. 2022 Nov 11;12:19298. doi: 10.1038/s41598-022-23966-4 (PMC9652473; doi:10.1038/s41598-022-23966-4)
Supplement: Supplementary file 4 — Supplementary Table S3. [file 41598_2022_23966_MOESM4_ESM.docx]

**Table S3**. The statistical parameters of the 4+3 OPLS_DA Model built on dataset including 5 classes (BMC, IP, CP, NB and MC)

| **Component** | **R^2^X** | **R^2^X(cum)** | **R^2^** | **R^2^(cum)** | **Q^2^** | **Q^2^(cum)** | **R^2^Y** | **R^2^Y(cum)** |
| --- | --- | --- | --- | --- | --- | --- | --- | --- |
| **Model** |  | 0.81 |  | ***0.593*** |  | ***0.517*** |  | 1 |
|  |  |  |  |  |  |  |  |  |
| **Predictive** |  | ***0.334*** |  | 0.593 |  | 0.517 |  | 1 |
| P1 | 0.149 | 0.149 | 0.15 | 0.15 | 0.11 | 0.11 | 0.23 | 0.234 |
| P2 | 0.079 | 0.228 | 0.19 | 0.337 | 0.17 | 0.278 | 0.3 | 0.531 |
| P3 | 0.052 | 0.279 | 0.15 | 0.484 | 0.13 | 0.408 | 0.23 | 0.76 |
| P4 | 0.055 | 0.334 | 0.11 | 0.593 | 0.11 | 0.517 | 0.24 | 1 |
|  |  |  |  |  |  |  |  |  |
| **Orthogonal in X (OPLS)** |  | ***0.475*** |  | 0 |  |  |  |  |
| O1 | 0.282 | 0.282 | 0 | 0 |  |  |  |  |
| O2 | 0.113 | 0.396 | 0 | 0 |  |  |  |  |
| O3 | 0.08 | 0.475 | 0 | 0 |  |  |  |  |

R^2^X - Fraction of X variation modelled in that component, using the X model.

R^2^ - Fraction of Y variation modelled in that component, using the X model.

Q^2^ - Fraction of Y variation predicted by the X model in that component, according to cross-validation.

R^2^Y - Fraction of the Y variation modelled in that component, using the Y model.

(cum) - Cumulative up to the specified component
