# Supplementary material for: Improved identification of pollution source attribution by using PAH ratios combined with multivariate statistics
Source: Sci Rep. 2022 Nov 11;12:19298. doi: 10.1038/s41598-022-23966-4 (PMC9652473; doi:10.1038/s41598-022-23966-4)
Supplement: Supplementary file 5 — Supplementary Table S4. [file 41598_2022_23966_MOESM5_ESM.docx]

**Table S4.** The statistical parameters of the 2+2 OPLS_DA Model built on dataset including 3 classes (BMC, IP, and CP)

| **Component** | **R2X** | **R2X(cum)** | **R2** | **R2(cum)** | **Q2** | **Q2(cum)** | **R2Y** | **R2Y(cum)** |
| --- | --- | --- | --- | --- | --- | --- | --- | --- |
| **Model** |  | 0.65 |  | ***0.675*** |  | ***0.618*** |  | 1 |
|  |  |  |  |  |  |  |  |  |
| **Predictive** |  | ***0.152*** |  | 0.675 |  | 0.618 |  | 1 |
| P1 | 0.0901 | 0.0901 | 0.395 | 0.395 | 0.345 | 0.345 | 0.558 | 0.558 |
| P2 | 0.0619 | 0.152 | 0.28 | 0.675 | 0.273 | 0.618 | 0.442 | 1 |
|  |  |  |  |  |  |  |  |  |
| **Orthogonal in X (OPLS)** |  | ***0.498*** |  | 0 |  |  |  |  |
| O1 | 0.281 | 0.281 | 0 | 0 |  |  |  |  |
| O2 | 0.217 | 0.498 | 0 | 0 |  |  |  |  |

R^2^X - Fraction of X variation modelled in that component, using the X model.

R^2^ - Fraction of Y variation modelled in that component, using the X model.

Q^2^ - Fraction of Y variation predicted by the X model in that component, according to cross-validation.

R^2^Y - Fraction of the Y variation modelled in that component, using the Y model.

(cum) - Cumulative up to the specified component
