# Supplementary material for: Improved identification of pollution source attribution by using PAH ratios combined with multivariate statistics
Source: Sci Rep. 2022 Nov 11;12:19298. doi: 10.1038/s41598-022-23966-4 (PMC9652473; doi:10.1038/s41598-022-23966-4)
Supplement: Supplementary file 6 — Supplementary Table S5. [file 41598_2022_23966_MOESM6_ESM.docx]

**Table S5**. Prediction test on the cross-validation set. Observations are colored based on YpredPS values: i. < 0.35 white (do not belong to the class); ii. 0.35-0.65 yellow (borderline); iii. > 0.65 green (belong to the class). The class attribution is based on the major value of YpredPS (pink).

| Primary | **YPredPS(BMC)** | **YPredPS(CP)** | **YPredPS(IPB)** | **Predicted_BMC** | **Predicted_CP** | **Predicted_IPB** |
| --- | --- | --- | --- | --- | --- | --- |
| TA04/0671a | 0.783969 | 0.0556796 | 0.160351 | 1 | 0 | 0 |
| TA04/0671b | 0.865472 | -0.252309 | 0.386837 | 1 | 0 | 0 |
| TA04/0671c | 0.76942 | 0.0576903 | 0.17289 | 1 | 0 | 0 |
| TA04/0672a | 0.455091 | 0.356533 | 0.188375 | 1 | 0 | 0 |
| TA04/0672b | 1.07575 | 0.0218837 | -0.097634 | 1 | 0 | 0 |
| TA04/0672c | 0.786306 | -0.0476532 | 0.261347 | 1 | 0 | 0 |
| TA04/0673a | 0.998715 | 0.122406 | -0.121121 | 1 | 0 | 0 |
| TA04/0673b | 0.884932 | 0.155319 | -0.0402512 | 1 | 0 | 0 |
| TA04/0673c | 0.832959 | 0.139471 | 0.0275696 | 1 | 0 | 0 |
| TA04/0674a | 1.07812 | 0.114109 | -0.192231 | 1 | 0 | 0 |
| TA04/0674b | 0.94305 | 0.0288066 | 0.028143 | 1 | 0 | 0 |
| TA04/0674c | 0.792089 | 0.158325 | 0.0495861 | 1 | 0 | 0 |
| TA04/0675a | 1.16279 | 0.00926949 | -0.172058 | 1 | 0 | 0 |
| TA04/0675b | 0.79726 | -0.147888 | 0.350628 | 1 | 0 | 0 |
| TA04/0675c | 1.23136 | -0.484598 | 0.253233 | 1 | 0 | 0 |
| TA04/0676a | 0.778309 | 0.142558 | 0.0791335 | 1 | 0 | 0 |
| TA04/0676b | 1.10559 | -0.0332301 | -0.072361 | 1 | 0 | 0 |
| TA04/0677a | 1.24514 | 0.0928993 | -0.338041 | 1 | 0 | 0 |
| TA04/0677b | 0.802987 | 0.157814 | 0.0391997 | 1 | 0 | 0 |
| TA04/0677c | 0.957453 | 0.0881942 | -0.0456474 | 1 | 0 | 0 |
| TA04/0678a | 1.74884 | -0.101584 | -0.647257 | 1 | 0 | 0 |
| TA04/0678b | 0.952409 | 0.102585 | -0.0549932 | 1 | 0 | 0 |
| TA04/0678c | 0.828691 | 0.21139 | -0.0400806 | 1 | 0 | 0 |
| TA04/0679a | 0.981369 | -0.303298 | 0.321929 | 1 | 0 | 0 |
| TA04/0679b | 0.799243 | 0.1455 | 0.0552572 | 1 | 0 | 0 |
| TA04/0679c | 0.706567 | 0.247892 | 0.0455411 | 1 | 0 | 0 |
| TA04/0680a | 0.501528 | 0.273241 | 0.225231 | 1 | 0 | 0 |
| TA04/0680b | 1.03816 | -0.164048 | 0.125886 | 1 | 0 | 0 |
| TA04/0680c | -0.351272 | 0.750221 | 0.60105 | 0 | 1 | 0 |
| TA04/0681a | 0.866105 | -0.0269503 | 0.160845 | 1 | 0 | 0 |
| TA04/0681b | 1.25067 | 0.0192395 | -0.269907 | 1 | 0 | 0 |
| TA04/0681c | 1.38748 | -0.327889 | -0.0595888 | 1 | 0 | 0 |
| TA04/0682a | 0.958575 | -0.229948 | 0.271373 | 1 | 0 | 0 |
| TA04/0682b | 0.780786 | -0.222826 | 0.44204 | 1 | 0 | 0 |
| TA04/0682c | 0.901178 | 0.00882058 | 0.0900013 | 1 | 0 | 0 |
| TA04/0683a | 0.683299 | 0.0957155 | 0.220986 | 1 | 0 | 0 |
| TA04/0683b | 0.838912 | -0.00871815 | 0.169806 | 1 | 0 | 0 |
| TA04/0683c | 0.93214 | 0.0929082 | -0.025048 | 1 | 0 | 0 |
| TA04/0683d | 1.10177 | 0.0412012 | -0.142975 | 1 | 0 | 0 |
| TA04/0684a | 0.817764 | 0.0603183 | 0.121917 | 1 | 0 | 0 |
| TA04/0684b | 0.846139 | 0.0452681 | 0.108593 | 1 | 0 | 0 |
| TA04/0684c | 0.790842 | 0.0842434 | 0.124914 | 1 | 0 | 0 |
| TA04/0685a | 0.761126 | 0.223584 | 0.0152901 | 1 | 0 | 0 |
| TA04/0685b | 0.861173 | 0.297257 | -0.15843 | 1 | 0 | 0 |
| TA04/0685c | 0.749899 | 0.503084 | -0.252983 | 1 | 0 | 0 |
| TA04/0685e | 0.872397 | 0.193517 | -0.0659141 | 1 | 0 | 0 |
| TA04/0685f | 0.792092 | 0.052261 | 0.155647 | 1 | 0 | 0 |
| TA04/0686a | 0.411646 | 0.282348 | 0.306006 | 1 | 0 | 0 |
| TA04/0686b | 0.91546 | -0.0111231 | 0.0956627 | 1 | 0 | 0 |
| TA04/0686c | 0.962313 | -0.0842893 | 0.121977 | 1 | 0 | 0 |
| TA04/0686d | 0.672664 | 0.10173 | 0.225606 | 1 | 0 | 0 |
| TA04/0687a | 0.733268 | 0.0379312 | 0.228801 | 1 | 0 | 0 |
| TA04/0687b | 0.650945 | -0.112922 | 0.461978 | 1 | 0 | 0 |
| TA04/0687c | 0.891669 | -0.0579025 | 0.166233 | 1 | 0 | 0 |
| TA04/0687d | 0.751508 | 0.0826597 | 0.165832 | 1 | 0 | 0 |
| TA04/0688a | 0.77829 | 0.17373 | 0.0479802 | 1 | 0 | 0 |
| TA04/0688b | 1.06592 | -0.452473 | 0.38655 | 1 | 0 | 0 |
| TA04/0688c | 0.997456 | -0.0585941 | 0.0611377 | 1 | 0 | 0 |
| TA04/0688d | 0.750807 | -0.0635591 | 0.312752 | 1 | 0 | 0 |
| TA04/0689a | 0.766525 | 0.27033 | -0.0368553 | 1 | 0 | 0 |
| TA04/0689b | 0.765361 | -0.0424851 | 0.277124 | 1 | 0 | 0 |
| TA04/0689c | 0.640069 | 0.114799 | 0.245132 | 1 | 0 | 0 |
| TA04/0689d | 0.85443 | 0.0329573 | 0.112612 | 1 | 0 | 0 |
| TA04/0690a | 0.779149 | 0.1766 | 0.0442502 | 1 | 0 | 0 |
| TA04/0690b | 0.496751 | 0.509828 | -0.00657964 | 0 | 1 | 0 |
| MP2_50 | -0.113607 | 0.945318 | 0.16829 | 0 | 1 | 0 |
| MP2_100 | -0.0245377 | 0.802653 | 0.221884 | 0 | 1 | 0 |
| MP3_50 | 0.10607 | 0.651632 | 0.242299 | 0 | 1 | 0 |
| MP4_50 | 0.101286 | 0.699303 | 0.199411 | 0 | 1 | 0 |
| MP6_50 | 0.33235 | 0.552322 | 0.115328 | 0 | 1 | 0 |
| MP14_50 | 0.0996529 | 0.690318 | 0.210029 | 0 | 1 | 0 |
| MP15_50 | 0.11312 | 0.570513 | 0.316368 | 0 | 1 | 0 |
| MP15_100 | 0.0349858 | 0.625947 | 0.339068 | 0 | 1 | 0 |
| MP018_50 | 0.243102 | 0.569681 | 0.187216 | 0 | 1 | 0 |
| MP17_50 | 0.277192 | 0.610319 | 0.11249 | 0 | 1 | 0 |
| MP19_50 | 0.0804577 | 0.741198 | 0.178344 | 0 | 1 | 0 |
| MP20_50 | 0.280499 | 0.597703 | 0.121798 | 0 | 1 | 0 |
| MP21_50 | 0.048004 | 0.756408 | 0.195588 | 0 | 1 | 0 |
| MP23_50 | 0.25371 | 0.601488 | 0.144802 | 0 | 1 | 0 |
| MP23_100 | -0.00428727 | 0.77776 | 0.226528 | 0 | 1 | 0 |
| MP24_50 | -0.0380825 | 0.783896 | 0.254186 | 0 | 1 | 0 |
| MP25_50 | -0.710542 | 1.46102 | 0.249521 | 0 | 1 | 0 |
| MP28_50 | -0.1486 | 0.956422 | 0.192177 | 0 | 1 | 0 |
| MP28_100 | -0.0476151 | 0.771344 | 0.276271 | 0 | 1 | 0 |
| TM_A_50 | 0.279478 | 0.765733 | -0.0452114 | 0 | 1 | 0 |
| TM_B_50 | 0.290425 | 0.840542 | -0.130968 | 0 | 1 | 0 |
| TM_C_50 | 0.209769 | 0.814589 | -0.024358 | 0 | 1 | 0 |
| TM_C_70 | 0.28993 | 0.701403 | 0.00866771 | 0 | 1 | 0 |
| TM_D_50 | 0.166583 | 0.875699 | -0.0422827 | 0 | 1 | 0 |
| TM_D_100 | 0.216106 | 0.700152 | 0.0837422 | 0 | 1 | 0 |
| TM_E_50 | 0.500939 | 0.332773 | 0.166288 | 1 | 0 | 0 |
| TM_E_100 | 0.343056 | 0.582624 | 0.0743201 | 0 | 1 | 0 |
| TM_F_50 | 0.518386 | 0.644487 | -0.162872 | 0 | 1 | 0 |
| BA01/0042a | 0.284179 | 0.273219 | 0.442601 | 0 | 0 | 1 |
| BA01/0042b | -0.0102413 | 0.0459449 | 0.964296 | 0 | 0 | 1 |
| BA01/0042 | 0.225942 | 0.436679 | 0.337379 | 0 | 1 | 0 |
| BA01/0044a | 0.192125 | -0.15597 | 0.963845 | 0 | 0 | 1 |
| BA01/0044b | -0.0111199 | -0.0500592 | 1.06118 | 0 | 0 | 1 |
| BA01/0047a | -0.0471732 | -0.0486055 | 1.09578 | 0 | 0 | 1 |
| BA01/0047 | 0.256053 | 0.145447 | 0.598501 | 0 | 0 | 1 |
| BA01/0047c | 0.00743321 | -0.156864 | 1.14943 | 0 | 0 | 1 |
| BA01/0051a | -0.0572055 | -0.360795 | 1.418 | 0 | 0 | 1 |
| BA01/0051 | 0.112986 | 0.203844 | 0.68317 | 0 | 0 | 1 |
| BA01/0051c | 0.166144 | -0.0955546 | 0.929411 | 0 | 0 | 1 |
| BA01/0053a | 0.0308701 | 0.00506803 | 0.964062 | 0 | 0 | 1 |
| BA01/0056a | 0.10261 | -0.065172 | 0.962562 | 0 | 0 | 1 |
| BA01/0059a | 0.127685 | -0.0948288 | 0.967144 | 0 | 0 | 1 |
| BA01/0061a0 | 0.223986 | 0.292435 | 0.483579 | 0 | 0 | 1 |
| BA01/0068a | 0.161116 | 0.312499 | 0.526385 | 0 | 0 | 1 |
| BA01/0068 | 0.0943273 | 0.164163 | 0.74151 | 0 | 0 | 1 |
| BA01/0069a | 0.205121 | 0.244254 | 0.550624 | 0 | 0 | 1 |
| BA01/0069 | 0.0190321 | 0.0825732 | 0.898395 | 0 | 0 | 1 |
| BA01/0069c | 0.37821 | 0.371842 | 0.249948 | 1 | 0 | 0 |
| BA01/0072/a | 0.269558 | 0.149274 | 0.581168 | 0 | 0 | 1 |
| BA01/0072/b | 0.243498 | 0.111169 | 0.645333 | 0 | 0 | 1 |
| BA01/0075a | 0.0895095 | 0.136324 | 0.774166 | 0 | 0 | 1 |
| BA01/0076a | 0.117278 | 0.139403 | 0.743319 | 0 | 0 | 1 |
| BA01/0076b | 0.0446112 | 0.0650868 | 0.890302 | 0 | 0 | 1 |
| BA01/0079a | 0.0125044 | -0.251732 | 1.23923 | 0 | 0 | 1 |
| BA01/0082a | 0.213352 | 0.136934 | 0.649714 | 0 | 0 | 1 |
| BA01/0082 | -0.0675124 | 0.103493 | 0.96402 | 0 | 0 | 1 |
| BA01/0082c | -0.0196199 | 0.0236872 | 0.995933 | 0 | 0 | 1 |
| BA01/0085a | 0.0570124 | 0.0736307 | 0.869357 | 0 | 0 | 1 |
| BA01/0085b | -0.0463974 | 0.0660242 | 0.980373 | 0 | 0 | 1 |
| BA01/0088a | 0.00214624 | 0.374777 | 0.623077 | 0 | 0 | 1 |
| BA01/0090a | 0.252206 | 0.205286 | 0.542508 | 0 | 0 | 1 |
| BA01/0124a | 0.393502 | 0.247031 | 0.359467 | 1 | 0 | 0 |
| BA01/0126a | 0.252818 | 0.104634 | 0.642548 | 0 | 0 | 1 |
| BA01/0128a | 0.463197 | 0.304779 | 0.232024 | 1 | 0 | 0 |
| BA01/0128b | 0.39513 | 0.207631 | 0.397239 | 0 | 0 | 1 |
| BA01/0130a | 0.365071 | 0.185814 | 0.449114 | 0 | 0 | 1 |
| BA01/0130b | 0.173338 | -0.0544419 | 0.881104 | 0 | 0 | 1 |
| tot |  |  |  | 4 | 11 | 35 |
